# Supplementary material for: miR-708-5p is elevated in bipolar patients and can induce mood disorder-associated behavior in mice
Source: EMBO Rep. 2025 Mar 10;26(8):2121–45. doi: 10.1038/s44319-025-00410-y (PMC12019553; doi:10.1038/s44319-025-00410-y)
Supplement: Supplementary file 1 — Appendix [file 44319_2025_410_MOESM1_ESM.pdf]

Appendix

miR-708-5p is elevated in bipolar patients and can induce mood disorder-associated behavior in mice

Carlotta Gilardi, Helena C. Martins, Brunno R. Levone, Alessandra Lo Bianco, Silvia Bicker, Pierre-Luc Germain, Fridolin Gross, Ayse Özge Sungur, Theresa M. Kisko, Frederike Stein, Susanne Meinert, Rainer K. W. Schwarting, Markus Wöhr, Udo Dannlowski, Tilo Kircher, and Gerhard Schratt

Table of Contents:

Appendix Table S1 .... page 2
Appendix Table S2 .... page 3
Appendix Figure S1 .... page 5
Appendix Figure S2 .... page 7
Appendix Figure S3 .... page 9
Appendix Figure S4 .... page 11
Appendix Figure S5 .... page 12
Appendix Figure S6 .... page 14

Appendix Tables

Appendix Table S1. Data from psychiatrically healthy female subjects (Control), or psychiatrically healthy female subjects with genetic predisposition for mood disorder (genetic risk – GR) or that suffered from childhood maltreatment (environmental risk – ER) (Vulnerability samples).

	**Control**	**ER**	**GR**	**P value**
n	18	17	18	N/A
**Sex**	Female	Female	Female	N/A
**Age ± *S.D.***	27.6 ± 7.1	34.9 ± 10.9	29.8 ± 9.5	0.11
**CTQ ± *S.D.***	29.7 ± 5.0	47.0 ± 10.4	30.2 ± 4.2	<0.0001

Subjects used for miR-708-5p expression analysis in healthy controls (Control), healthy subjects with a history of Childhood Maltreatment (ER), healthy control with genetic risk (GR). One-way ANOVA was performed to evaluate significant differences between groups. *S.D.* standard deviation, CTQ: Childhood Maltreatment Questionnaire.

Appendix Table S2. Sequence of all oligos used for cloning.

Name of the sequence	Sequence (5'->3')	Construct
Chimeric miR-708 hairpin A_Forward	GTACAGCTGTTGACAGTGAGCGACAAGGAGCTT	hp708
Chimeric miR-708 hairpin A_Reverse	GATTGTAAGCTCCTTGTCGCTCACTGTCAACAGCT	hp708
Chimeric miR-708 hairpin B_Forward	TCTAGCTGGGTGTGAAGCCACAGATGGCCCAGCTA	hp708
Chimeric miR-708 hairpin B_Reverse	AGTAAGCTCCTTGCTGCCTACTGCCTCGGAA	hp708
Chimeric miR-708 hairpin C_Forward	TTACTCTAGCTGGGCCATCTGTGGCTTCACACCCA	hp708
Chimeric miR-708 hairpin C_Reverse	AGCTTTCCGAGGCAGTAGGCAGCAAGGAG	hp708
Chimeric control hairpin A_Forward	TGTACAGCTGTTGACAGTGAGCGACAACCTTGTG	hpCTL
Chimeric control hairpin A_Reverse	AAGGACCACAAGGTTGTCGCTCACTGTCAACAGC	hpCTL
Chimeric control hairpin B_Forward	GTCCTTAGGTGCGTGTGAAGCCACAGATGGCGC	hpCTL
Chimeric control hairpin B_Reverse	GGTTTAGGTGCGCCATCTGTGGCTTCACACGCACCT	hpCTL
Chimeric control hairpin C_Forward	ACCTAAACCACAAGGTTGCTGCCTACTGCCTCGGA	hpCTL
Chimeric control hairpin C_Reverse	AAGCTTTCCGAGGCAGTAGGCAGCAACCTTG	hpCTL
miR-708-5p six TDMD sequence	TGTACATATAACTAGTTGCATTGTCGATTCATCCCAGCTA GATTGCAATAGCTCCTTGACTCCCAGCTAGATTGCAATA GCTCCTTCAGTCCCAGCTAGATTGCAATAGCTCCTTGAC TCCCAGCTAGATTGCAATAGCTCCTTACGTCCCAGCTAG ATTGCAATAGCTCCTTGTCACCCAGCTAGATTGCAATAG CTCCTTGATCGTAGCTAAGCTTGTCGAC	sp708
Control six TDMD sequence	TGTACATATAACTAGTTGCATTGTCGATTCATTGCTACAT GGTCGGAACGGTCTGACTATCTTCGAGGTCGGTTTCTT ACCGCTCACTTAGTGGTCCGTCGGTGATGACTATCATG	spCTL

	CTGGTCACTTTGGCGGACGTCTCTATGAGGTCTATGGTCCGGGTCATCTTGACAGGTCAGGCGTTCGTGATCGTAGCTAAGCTTGTCGAC	
**PBS miR708-fw**	ACTAGTCCCAGCTAGATTGTAAGCTCCTTCTCCCAGCTAGATTGTAAGCTCCTT	pMIR-GLO-2xPBS miR-708-5p
**PBS miR708-rv**	AAGCTTAAGGAGCTTACAATCTAGCTGGGagAAGGAGCTTACAATCTAGCTGGG	pMIR-GLO-2xPBS miR-708-5p
**Nnat 3'-UTR fw**	TAAGCAGAGCTCCCCCAGCTCCCAGCCct	pMiR-GLO-Nnat3'UTR-WT
**feNnat 3'-UTR rv**	TAAGCAGTCGACTTTTGGTGCACCCCCACT	pMiR-GLO-Nnat3'UTR-WT
**Nnat 3'-UTR mutagenesis fw**	CGAGCTACATTgTACGCTGCTGGAGACAGGGACCACCTC	pMiR-GLO-Nnat3'UTR-mt
**Nnat 3'-UTR mutagenesis rv**	AGCAGCGTAcAATGTAGCTCGGGAGACACTACTAATGCACACTT	pMiR-GLO-Nnat3'UTR-mt

Appendix Figures

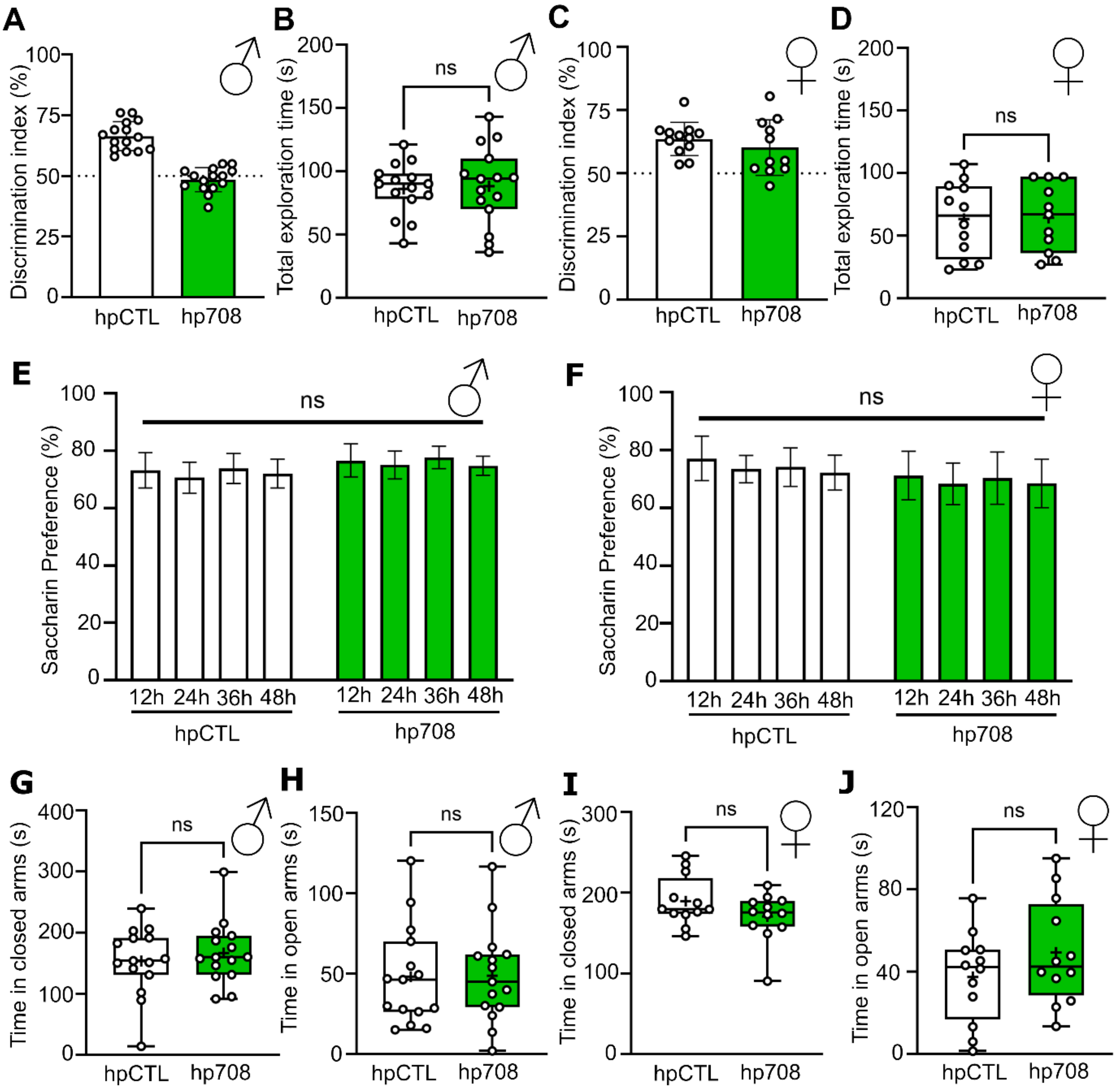

Appendix Figure S1. Further behavioral characterization of miR-708-5p overexpression in mice. Refer to Fig. 3.

A. Discrimination index calculated as time spent exploring novel object / time spent exploring novel and familiar objects for male mice male mice injected with the indicated rAAV (hpCTL or hp708, n=15 each) in the short-term memory NOR session (Fig. 3D). Data are represented as scattered dot plots with bar, mean ± SD.

B. Time (s) male mice injected with the indicated rAAV (hpCTL or hp708, n=15 each) explored the familiar (F) and novel (N) object in the short-term memory NOR session (Fig. 3D). Data are represented as box plot with whiskers and data points (+: mean, line: median; whiskers: minimum and maximum values). Unpaired t-test, ns, p=0.8207.

C. Discrimination index calculated as time spent exploring novel object / time spent exploring novel and familiar objects for female mice male mice injected with the indicated rAAV (hpCTL n= 12, or hp708, n=11) in the short-term memory NOR session (Fig. 3E). Data are represented as scattered dot plots with bar, mean ± SD.

D. Novel object recognition Test with 5 minutes break in between familiarization and novelty testing. Time (s) female mice injected with the indicated rAAV (hpCTL n= 12, or hp708, n=11) explored the familiar (F) and novel (N) object in the short-term memory NOR session (Fig. 3E). Data are represented as box plot with whiskers and data points (+: mean, line: median; whiskers: minimum and maximum values). Unpaired t-test, ns, p=0.9257.

E. Cumulative Saccharin Preference (%) of male mice injected with the indicated rAAV (hpCTL or hp708, n=10 each). Data are represented as bar plot and mean ± SD. Two-way RM ANOVA: Timepoint x Group, ns, p=0.6645; Timepoint, **, p=0.0067; Group, ns, p=0.1088. Šídák's post hoc test, hpCTL *vs* hp708: 12h, ns, p=0.6407; 24h, ns, p=0.2698; 36h, ns, p=0.3400; 48h, ns, p=0.5744.

F. Cumulative Saccharin Preference (%) of female mice injected with the indicated rAAV (hpCTL n= 12, or hp708, n=11). Data are represented as bar plot and mean ± SD. Two-way RM ANOVA: Timepoint x Group, ns, p=0.7385; Timepoint, *, p=0.0489; Group, ns, p=0.1098. Šídák's post hoc test, hpCTL *vs* hp708: 12h, ns, p=0.3540; 24h, ns, p=0.2635; 36h, ns, p=0.7392; 48h, ns, p=0.6978.

G. and **H.** Time (s) male mice injected with the indicated rAAV (hpCTL or hp708, n=15 each) spent in closed (G) or open arm (H) of the Elevated Plus Maze. Data are represented as box plot with whiskers and data points (+: mean, line: median; whiskers: minimum and maximum values). Unpaired t-test, open arms, ns, p=0.5091; closed arms, ns, p=0.9516.

I. and **J.** Time (s) female mice injected with the indicated rAAV (hpCTL n= 12, or hp708, n=11) spent in closed (I) or open arm (J) of the Elevated Plus Maze. Data are represented as box plot with whiskers and data points (+: mean, line: median; whiskers: minimum and maximum values). Unpaired t-test, closed arms, ns, p=0.1412; open arms, ns, p=0.2364.

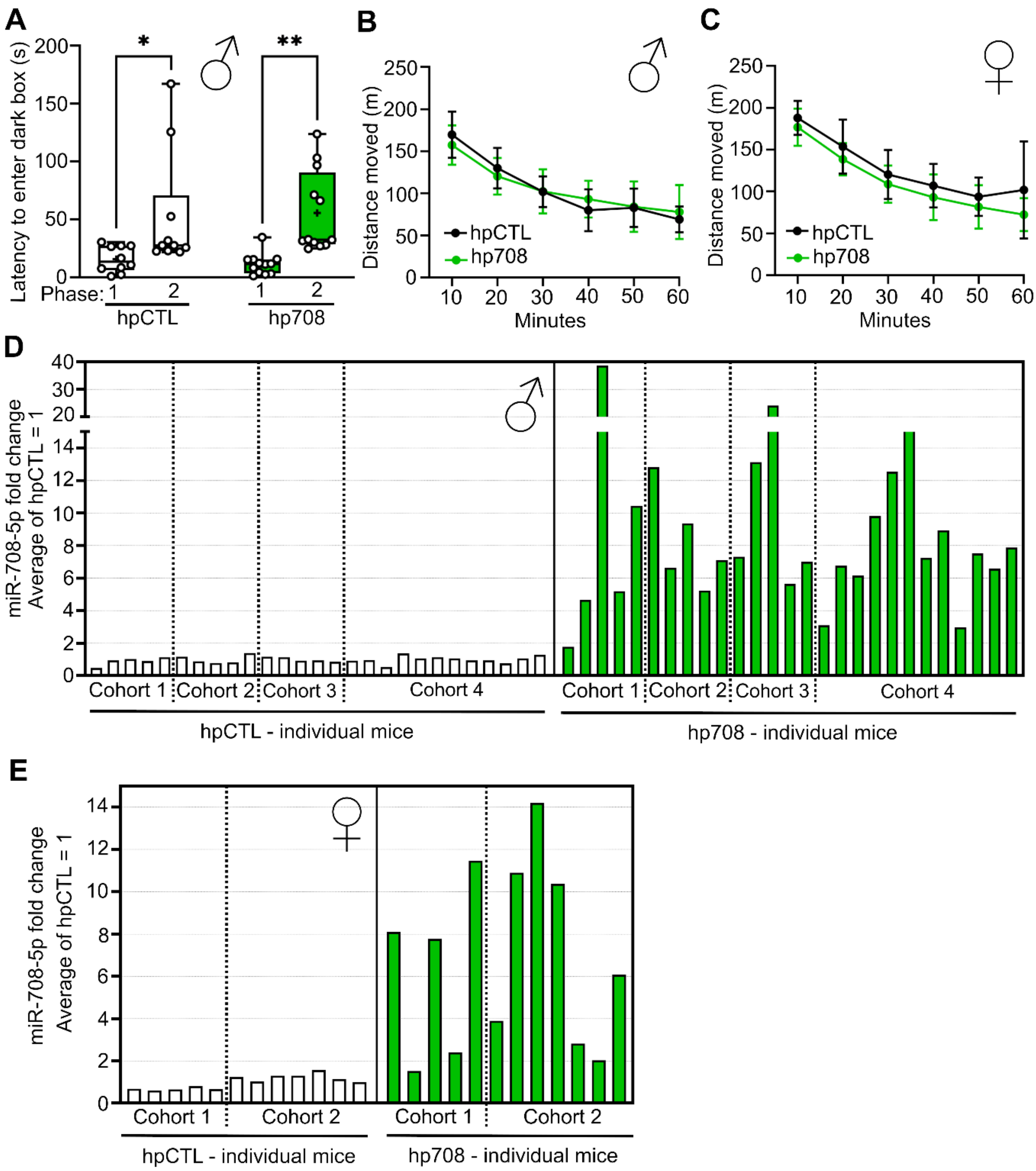

Appendix Figure S2. Further characterization of miR-708-5p overexpression in mice. Refer to Fig. 3.

A. Passive Avoidance Test. Latency (s) male mice injected with the indicated rAAV (hpCTL n= 10, or hp708, n=12) to enter the dark box in Phase1 and Phase 2. Data are represented as box plot with whiskers and data points (+: mean, line: median; whiskers: minimum and maximum values). Two-way RM ANOVA: Phase x Group, ns, p=0.6815; Phase, ***, p=0.0003; Group, ns, p=0.9451. Šídák's post hoc test, Phase 1 *vs* Phase 2: hpCTL, *, p=0.0265; hp708, **, p=0.0036.

B. Total Distance travelled (cm) in the open field (over 60 min) by male mice injected with the indicated rAAV (hpCTL n=14, or hp708, n=12). Data are represented as XY axis with Mean ± SD.

C. Total Distance travelled (cm) in the open field (over 60 min) by female mice injected with the indicated rAAV (hpCTL n= 12, or hp708, n=11). Data are represented as XY axis with Mean ± SD.

D. and **E.** miR-708-5p qPCR analysis of total RNA isolated from mouse hippocampi upon miR-708-5p overexpression after behavioral characterization. Each bar corresponds to one animal, for male (D) or female (E) mice. Data are represented as a nested bar graph, with the individual values being normalized to the average of the hpCTL group (set at 1).

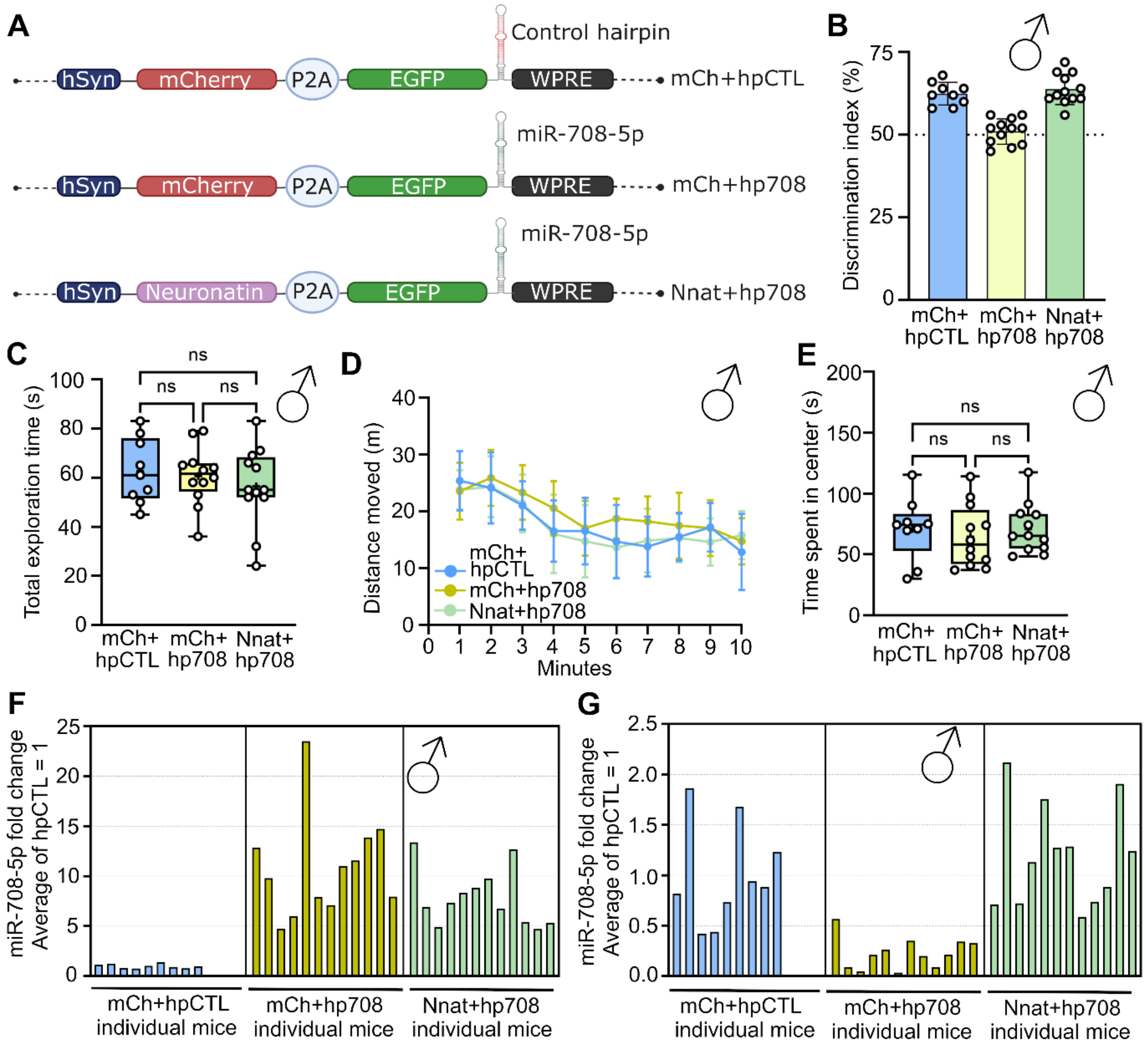

Appendix Figure S3. Further characterization of Neuronatin rescue of miR-708-5p overexpression experiment. Refer to Fig. 5.

A. Schematic representation of the three rAAV constructs used in the rescue experiment.

B. Discrimination index (Fig. 5A) calculated as time spent exploring novel object / time spent exploring novel and familiar objects in the Novel object recognition test (short-term memory session) by male mice injected with mCherry-hpCTL (mCh+hpCTL, n=9), mCherry-hp708 (mCh+hp708, n=12), or Nnat+hp708 (n=12) viruses. Data are represented as scattered dot plots with bar, mean $\pm$ SD.

C. Time (s) male mice injected with mCh+hpCTL (n=9), mCh+hp708 (n=12), or Nnat-P2A-hp708 (n=12) viruses explored the familiar (F) and novel (N) objects in the Novel object recognition test (short-term memory session, Fig. 5A). Data are represented as box plot with whiskers and data points (+: mean, line: median; whiskers: minimum and maximum values). One-way ANOVA, mCh+hpCTL *vs*

mCh+hp708, ns, p=0.9439; mCh+hpCTL *vs* Nnat+hp708, ns, p=0.5747; mCh+hp708 *vs* Nnat+hp708, p=0.7396.

D. Open Field Test. Total Distance travelled (cm) by male mice injected with mCh+hpCTL (n=9), mCh+hp708 (n=12), or Nnat+hp708 (n=12) viruses in the open field (over 10 min). Data are represented as XY axis with Mean $\pm$ SD.

E. Time (s) male mice injected with mCherry-P2A-hpCTL (n=9), mCherry-P2A-hp708 (n12), or Nnat-P2A-hp708 (n=12) viruses spent exploring the center of the open field arena. Data are represented as box plot with whiskers and data points (+: mean, line: median; whiskers: minimum and maximum values). One-way ANOVA, mCh+hpCTL *vs* mCh+hp708, ns, p=0.8167; mCh+hpCTL *vs* Nnat+hp708, ns, p>0.9999; mCh+hp708 *vs* Nnat+hp708, p=0.7917.

F. miR-708-5p qPCR analysis of total RNA isolated from mouse hippocampi upon miR-708-5p overexpression with or without the co-overexpression of *Nnat* after behavioral characterization. Each bar corresponds to one animal. Data are represented as nested bar graphs.

G. *Nnat* qPCR analysis of total RNA isolated from mouse hippocampi upon miR-708-5p overexpression with or without the co-overexpression of *Nnat* after behavioral characterization. Each bar corresponds to one animal. Data are represented as nested bar graphs.

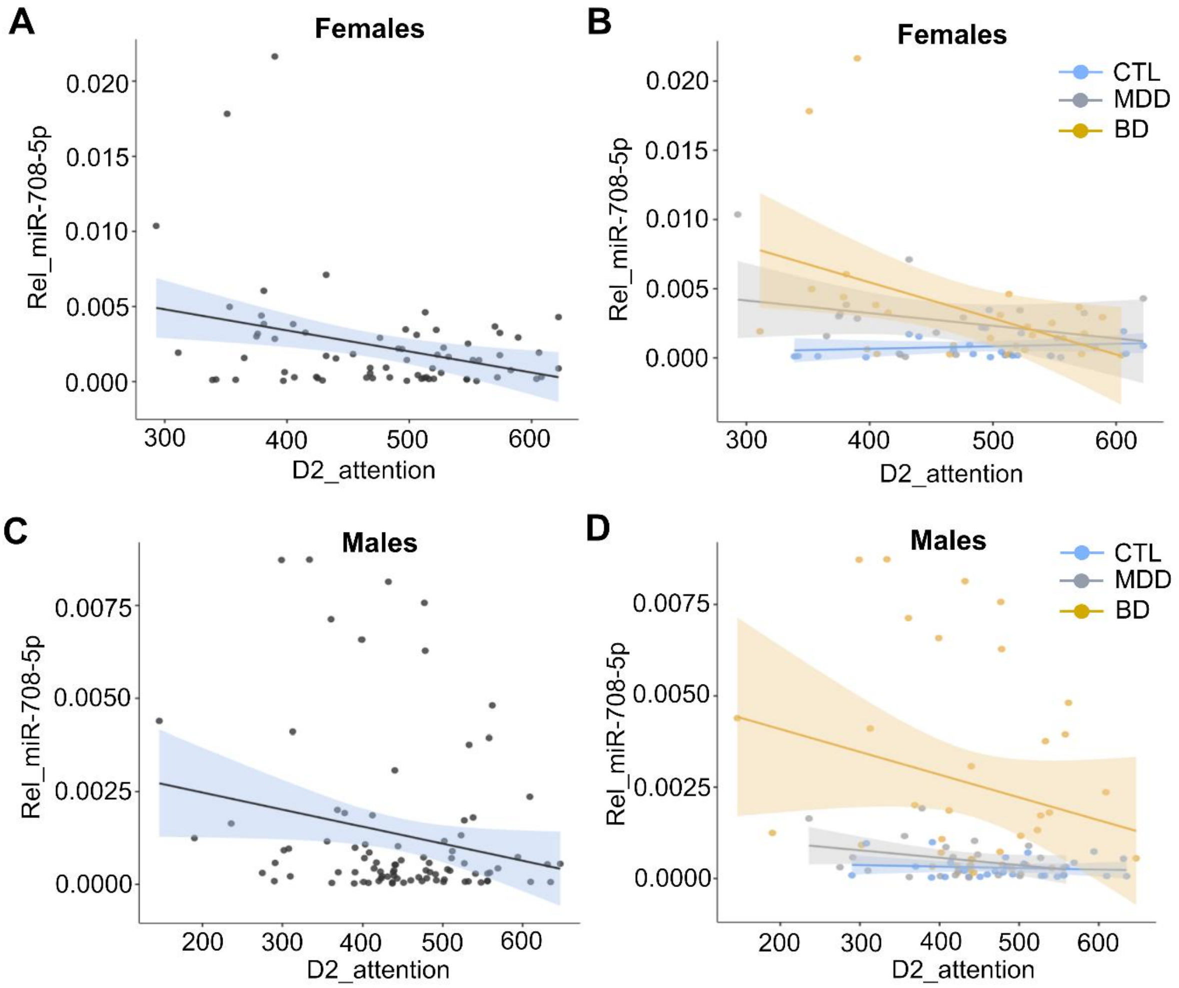

Appendix Figure S4. Correlation between attention (D2 test) and miR-708-5p in males and females, and by group. Refer to Fig. 6.

A. Pearson correlation plot of miR-708-5p peripheral levels and attention (d2 test) in all female participants (healthy control (HC) + MDD + BD, n=70), ***, p=0.006, r=-0.324.

B. Pearson correlation plot of miR-708-5p peripheral levels and attention (d2 test) in all female participants (healthy control (HC) + MDD + BD, n=70), split by disorder group.

C. Pearson correlation plot of miR-708-5p peripheral levels and attention (d2 test) in all male participants (healthy control (HC) + MDD + BD, n=92), *, p=0.047, r=-0.221.

D. Pearson correlation plot of miR-708-5p peripheral levels and attention (d2 test) in all male participants (healthy control (HC) + MDD + BD, n=92), split by disorder group.

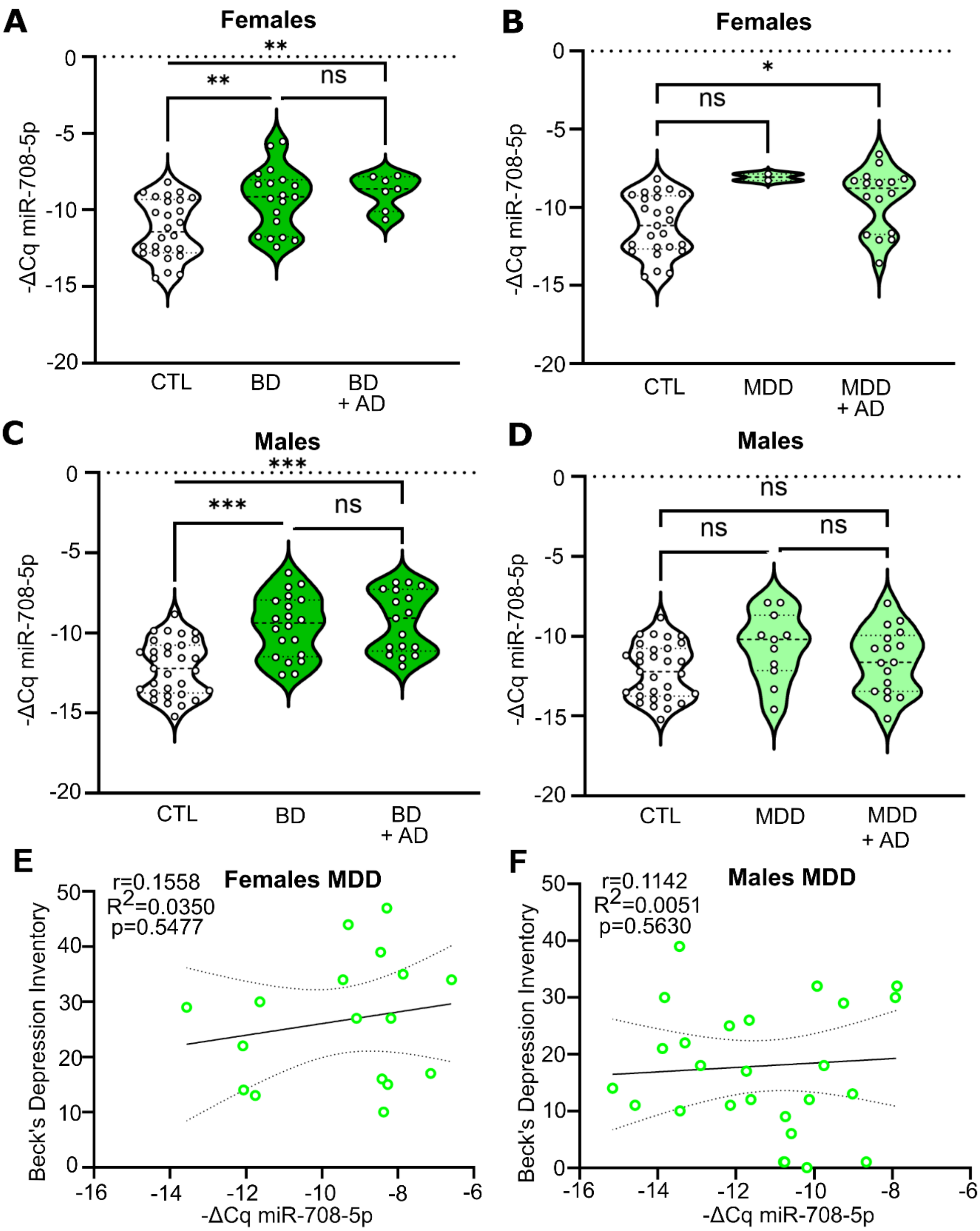

Appendix Figure S5. miR-708-5p expression levels in BD and MDD groups by the use (or not) of antidepressants, and correlation with the Beck's Depression Inventory score. Refer to Fig. 6.

A. miR-708-5p qPCR analysis of total RNA isolated from PBMCs of female subjects (control, n=26; BD, n= 19; BD+AD, n=7). Kruskal Wallis Test, Post hoc Dunn's Test: Control vs BD: **, p=0.0091, Control vs BD+AD: **, p=0.0089, BD vs BD+AD: ns, p>0.9999. Data are presented as violin plots with median, quartiles and data points.

B. miR-708-5p qPCR analysis of total RNA isolated from PBMCs of female subjects (control, n=26; MDD, n= 2; MDD+AD, n=16). Kruskal Wallis Test, Post hoc Dunn's: Control vs MDD: ns, p=0.0532, Control vs MDD+AD: ns, p=0.0295, MDD vs MDD+AD: ns, p=0.6661. Data are presented as violin plots with median, quartiles and data points.

C. miR-708-5p qPCR analysis of total RNA isolated from PBMCs of male subjects (control, n=31; BD, n= 20; BD+AD, n=17). Kruskal Wallis Test, Post hoc Dunn's Test: Control vs BD: ***, p=0.0004, Control vs BD+AD: ***, p=0.0002, BD vs BD+AD: ns, p>0.9999. Data are presented as violin plots with median, quartiles and data points.

D. miR-708-5p qPCR analysis of total RNA isolated from PBMCs of male subjects (control, n=31; MDD, n= 11; MDD+AD, n=17). One-way ANOVA, Post hoc Tukey's test: Control vs MDD: ns, p=0.0626, Control vs MDD+AD: ns, p=0.5181, MDD vs MDD+AD: ns, p=0.4368. Data are presented as violin plots with median, quartiles and data points.

E. Correlation plot between the peripheral levels of miR-708-5p in female MDD samples (n=18) and Beck's Depression Inventory. Spearman correlation coefficient with two-tailed analysis. Data are presented as XY tables.

F. Correlation plot between the peripheral levels of miR-708-5p in male MDD samples (n=28) and Beck's Depression Inventory. Spearman correlation coefficient with two-tailed analysis. Data are presented as XY tables.

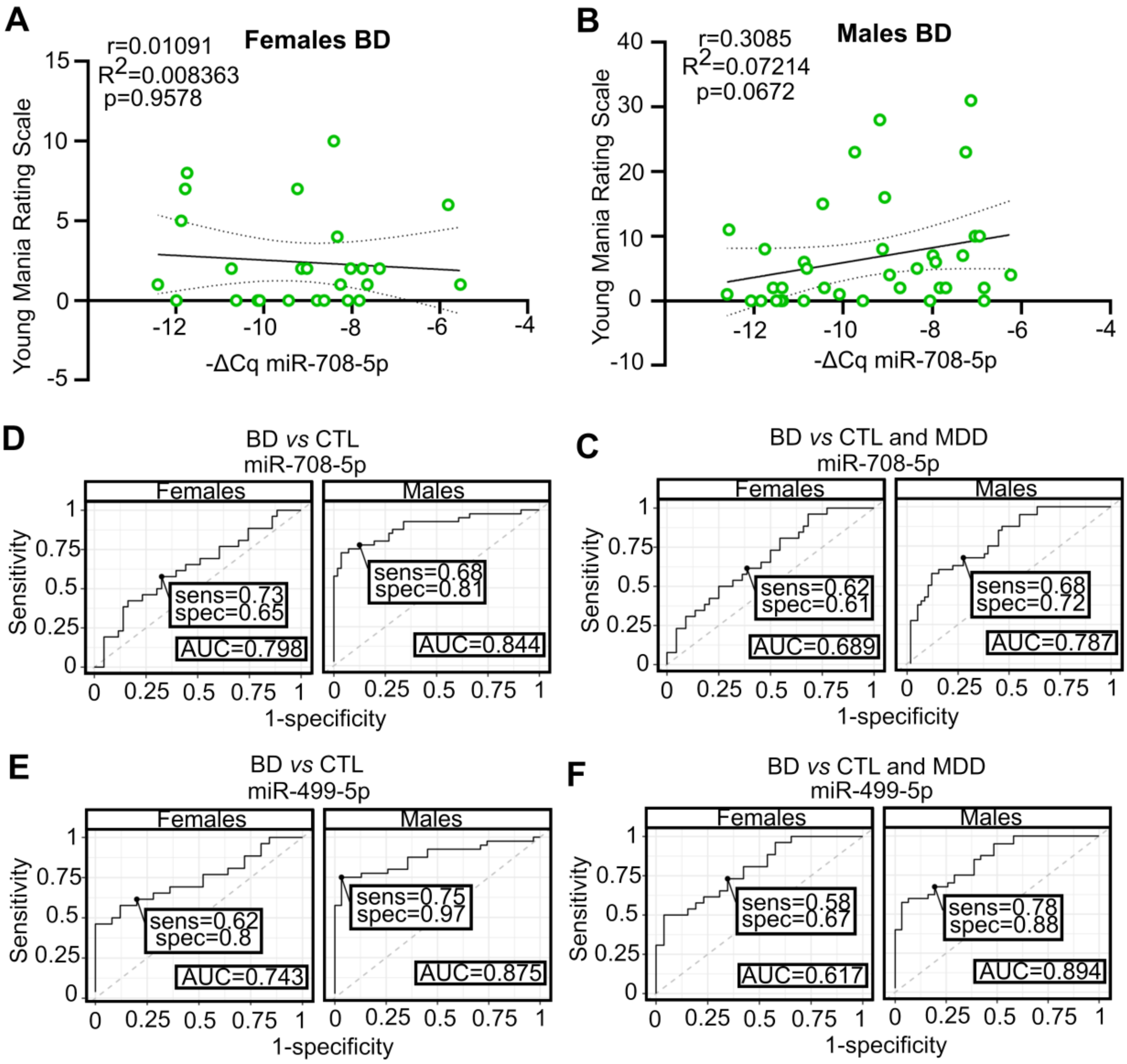

Appendix Figure S6. miR-708-5p expression levels and correlation with the Young Mania Rating Scale score and ROC for BD *vs* CTL or *vs* CTL and MDD for either miR-708-5p or miR-499-5p. Refer to Fig. 6.

A. Correlation plot between the peripheral levels of miR-708-5p in female BD samples (n=26) and Young Mania Rating Scale. Spearman correlation coefficient with two-tailed analysis. Data are presented as XY tables.

B. Correlation plot between the peripheral levels of miR-708-5p in male BD samples (n=37) and Young Mania Rating Scale. Spearman correlation coefficient with two-tailed analysis. Data are presented as XY tables.

C. and **D.** ROC curves of miR-708-5p expression in female (left) and male (right) subjects to discriminate BD *vs* controls (C) and BD *vs* controls and MDD samples (D). The indicated thresholds are the closest point to the optimal (i.e., top-left).

E. and **F.** ROC curves of miR-499-5p expression in female (left) and male (right) subjects to discriminate BD *vs* controls (E) and BD *vs* controls and MDD samples (F). The indicated thresholds are the closest point to the optimal (i.e. top-left).
