# Supplementary figures and images for: miR-708-5p is elevated in bipolar patients and can induce mood disorder-associated behavior in mice
Source: EMBO Rep. 2025 Mar 10;26(8):2121–45. doi: 10.1038/s44319-025-00410-y (PMC12019553; doi:10.1038/s44319-025-00410-y)

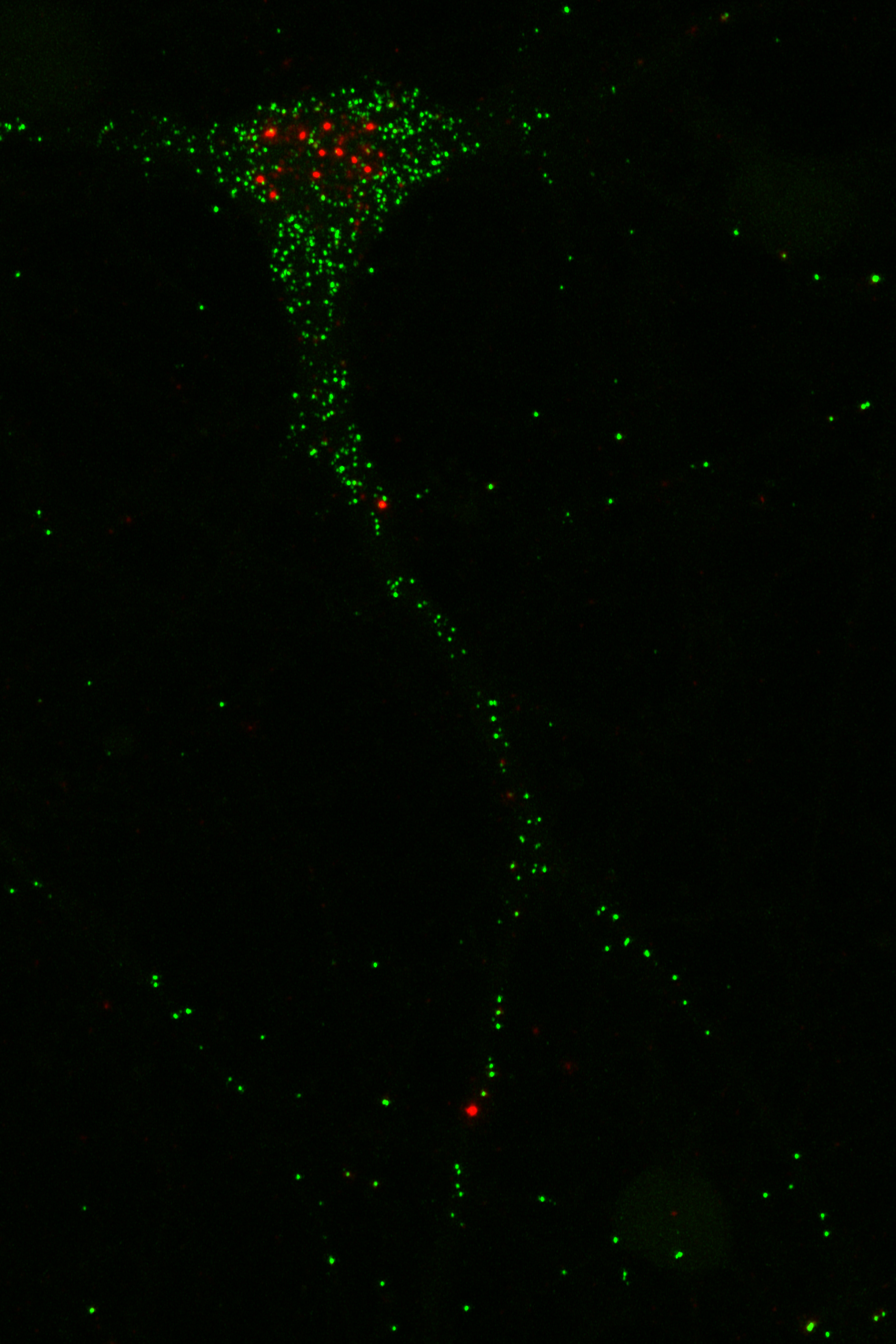

Supplement: Supplementary file 4 — Source data Fig. 2 [file 44319_2025_410_MOESM4_ESM.zip › 2B/2B left panel - cropped.tif]

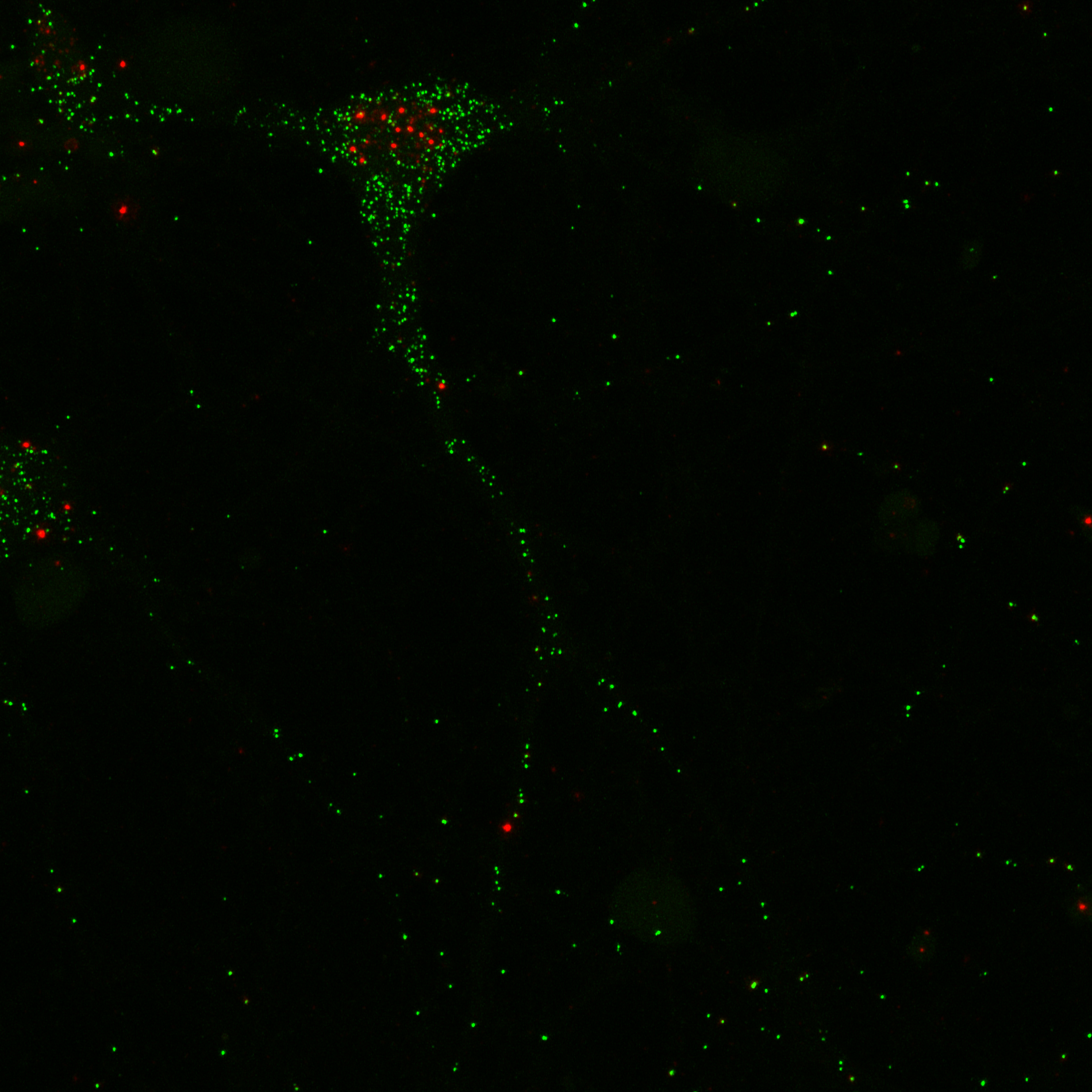

Supplement: Supplementary file 4 — Source data Fig. 2 [file 44319_2025_410_MOESM4_ESM.zip › 2B/2B left panel - full.tif]

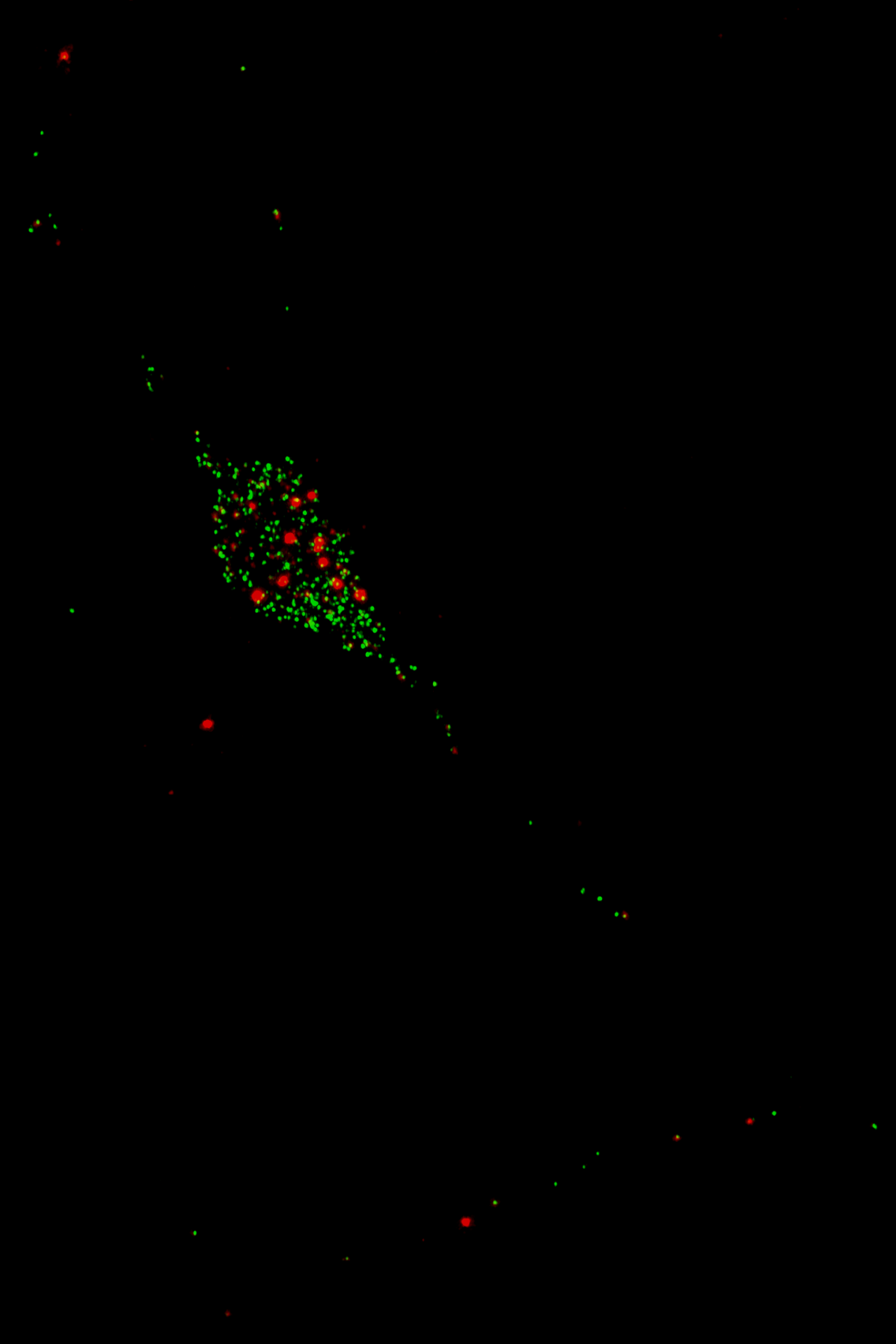

Supplement: Supplementary file 4 — Source data Fig. 2 [file 44319_2025_410_MOESM4_ESM.zip › 2B/2B right panel - cropped.tif]

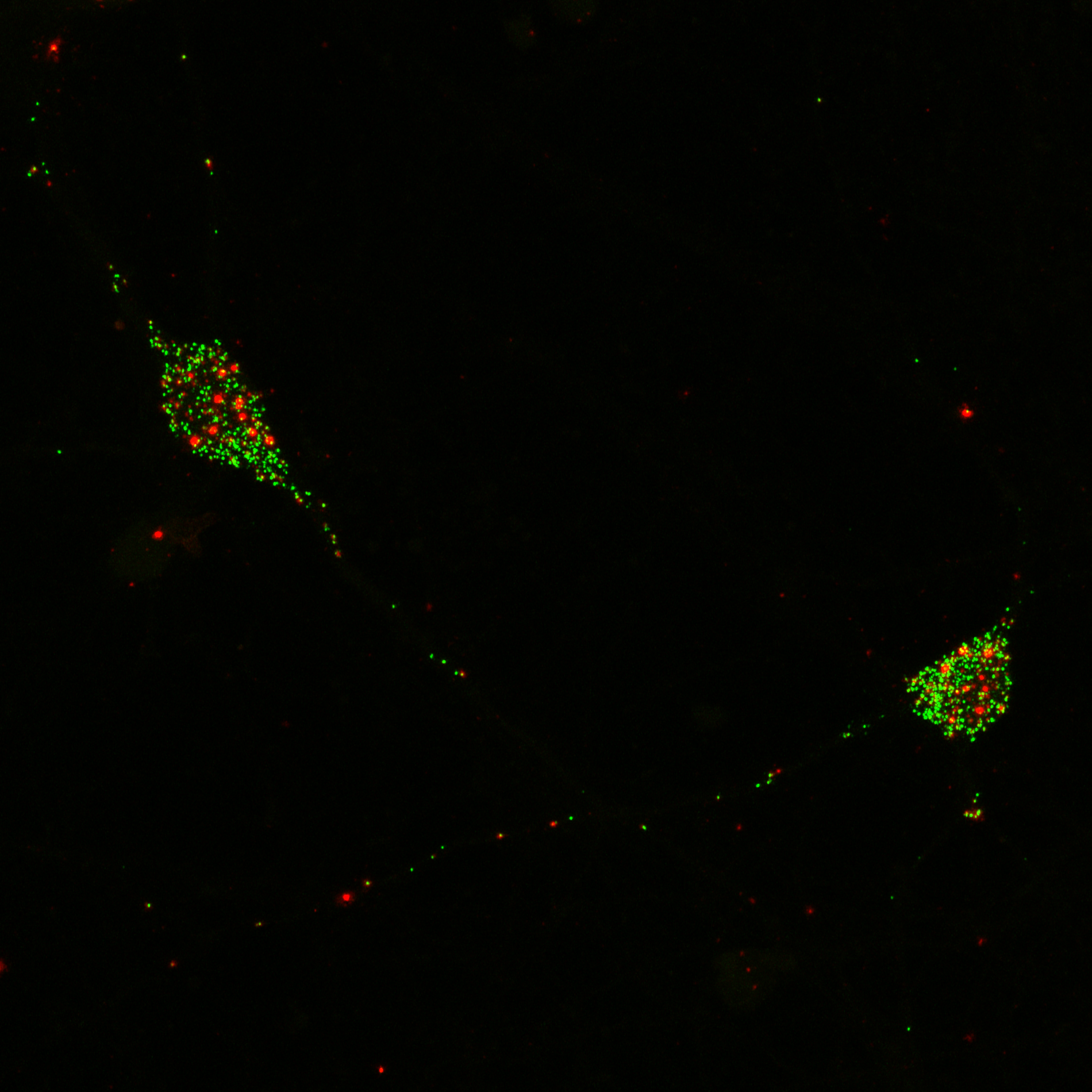

Supplement: Supplementary file 4 — Source data Fig. 2 [file 44319_2025_410_MOESM4_ESM.zip › 2B/2B right panel - full.tif]

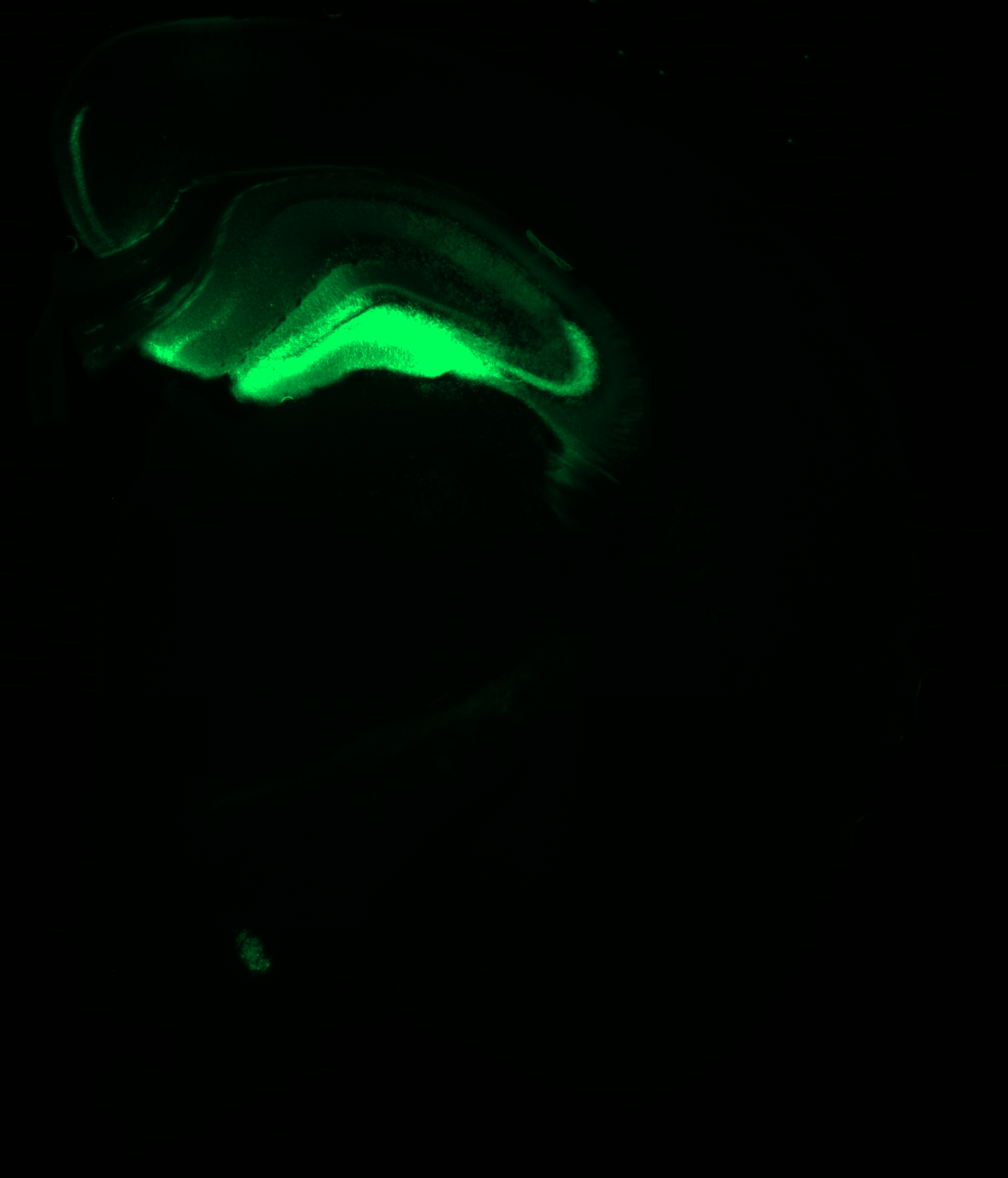

Supplement: Supplementary file 5 — Source data Fig. 3 [file 44319_2025_410_MOESM5_ESM.zip › 3B/Dorsal - GFP.tif]

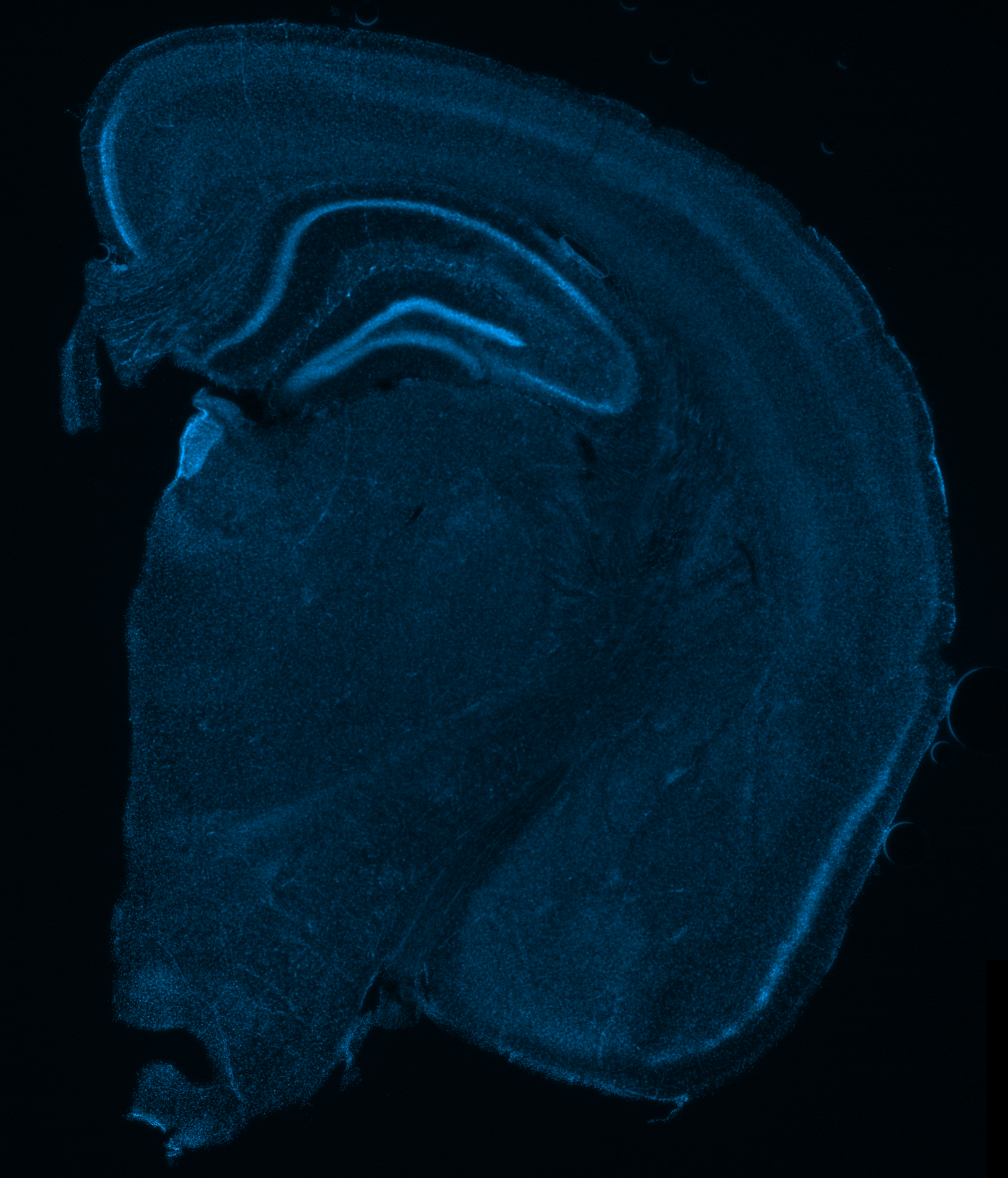

Supplement: Supplementary file 5 — Source data Fig. 3 [file 44319_2025_410_MOESM5_ESM.zip › 3B/Dorsal - Hoechst.tif]

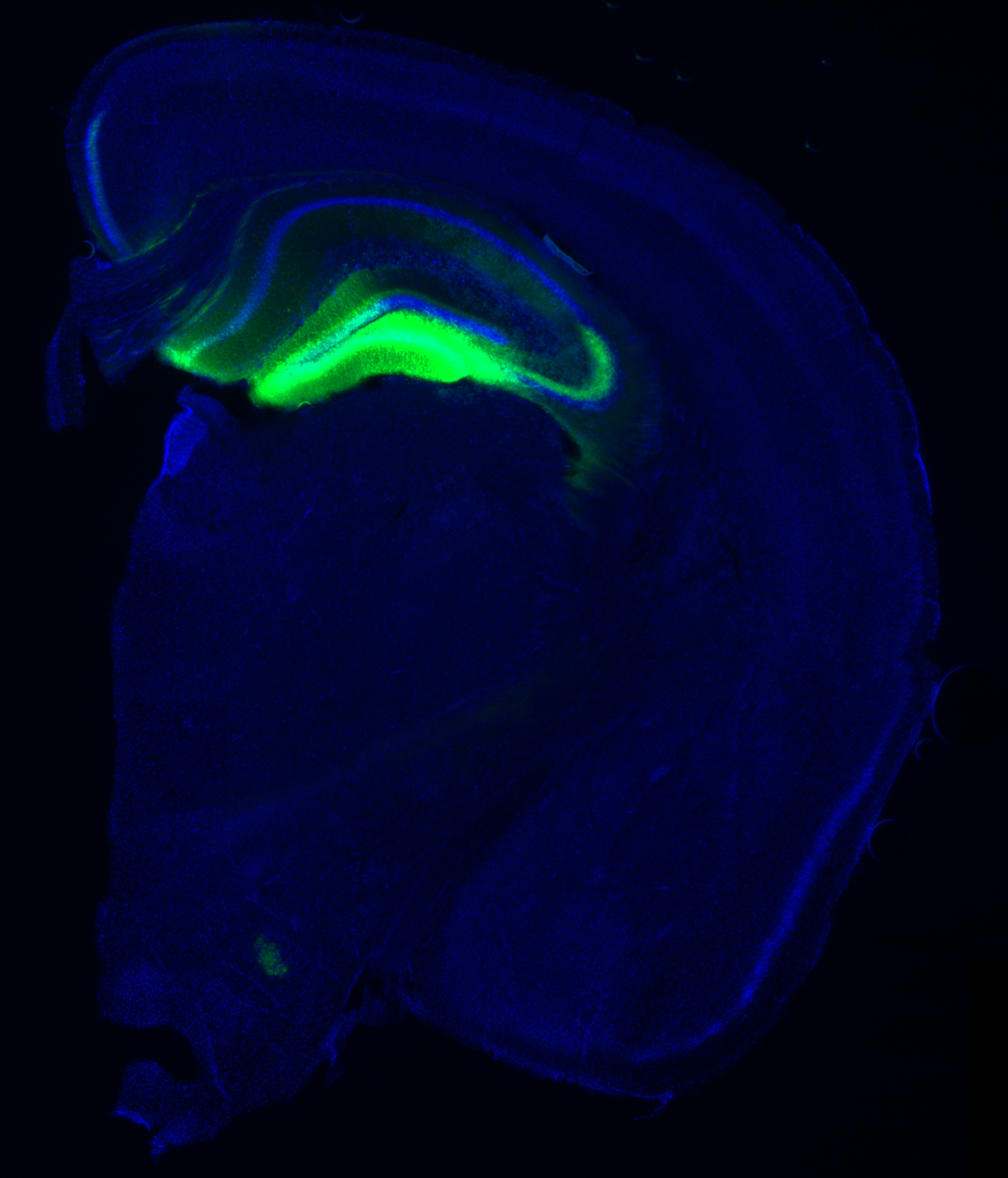

Supplement: Supplementary file 5 — Source data Fig. 3 [file 44319_2025_410_MOESM5_ESM.zip › 3B/Dorsal - Merge.tif]

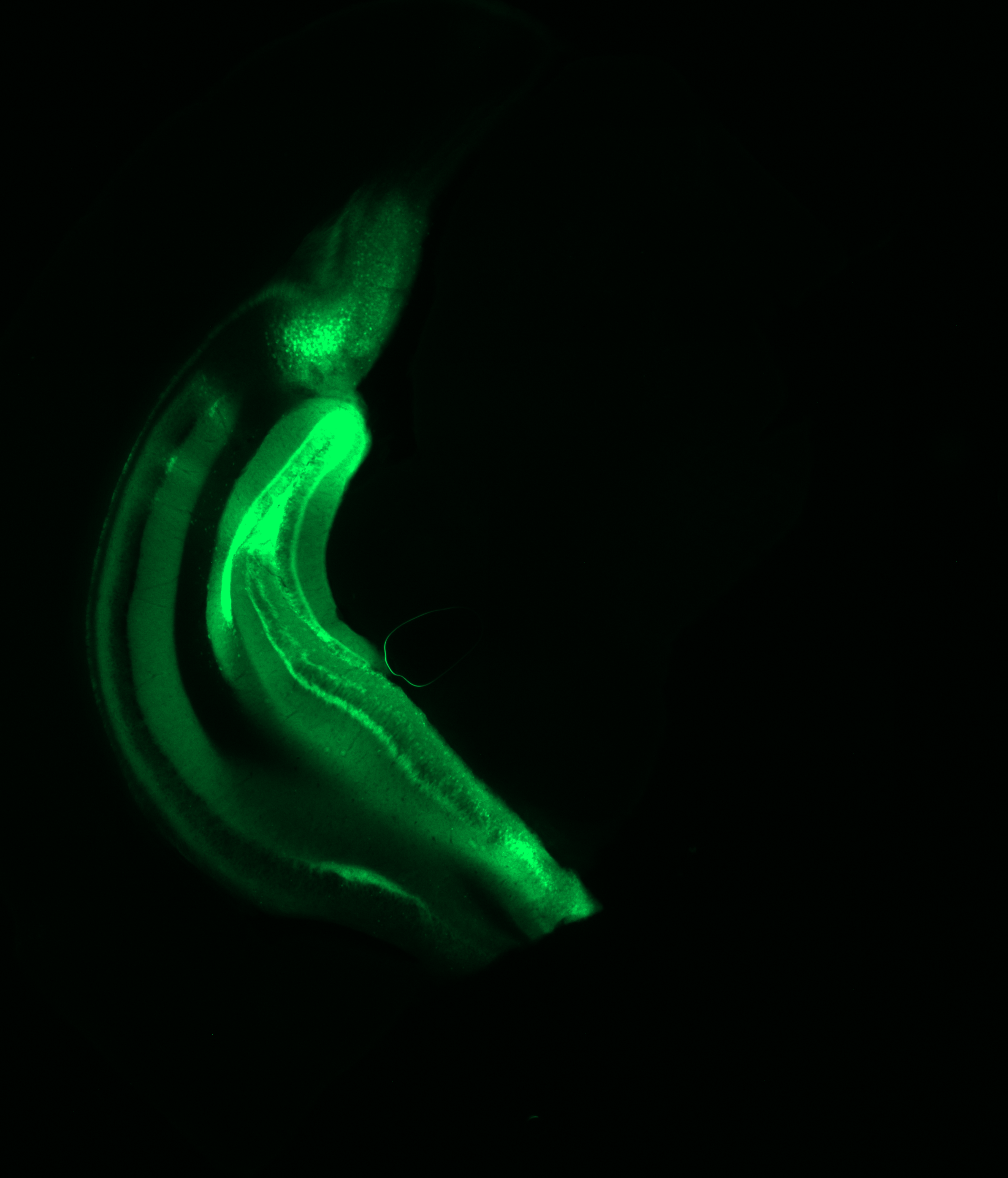

Supplement: Supplementary file 5 — Source data Fig. 3 [file 44319_2025_410_MOESM5_ESM.zip › 3B/Ventral - GFP.tif]

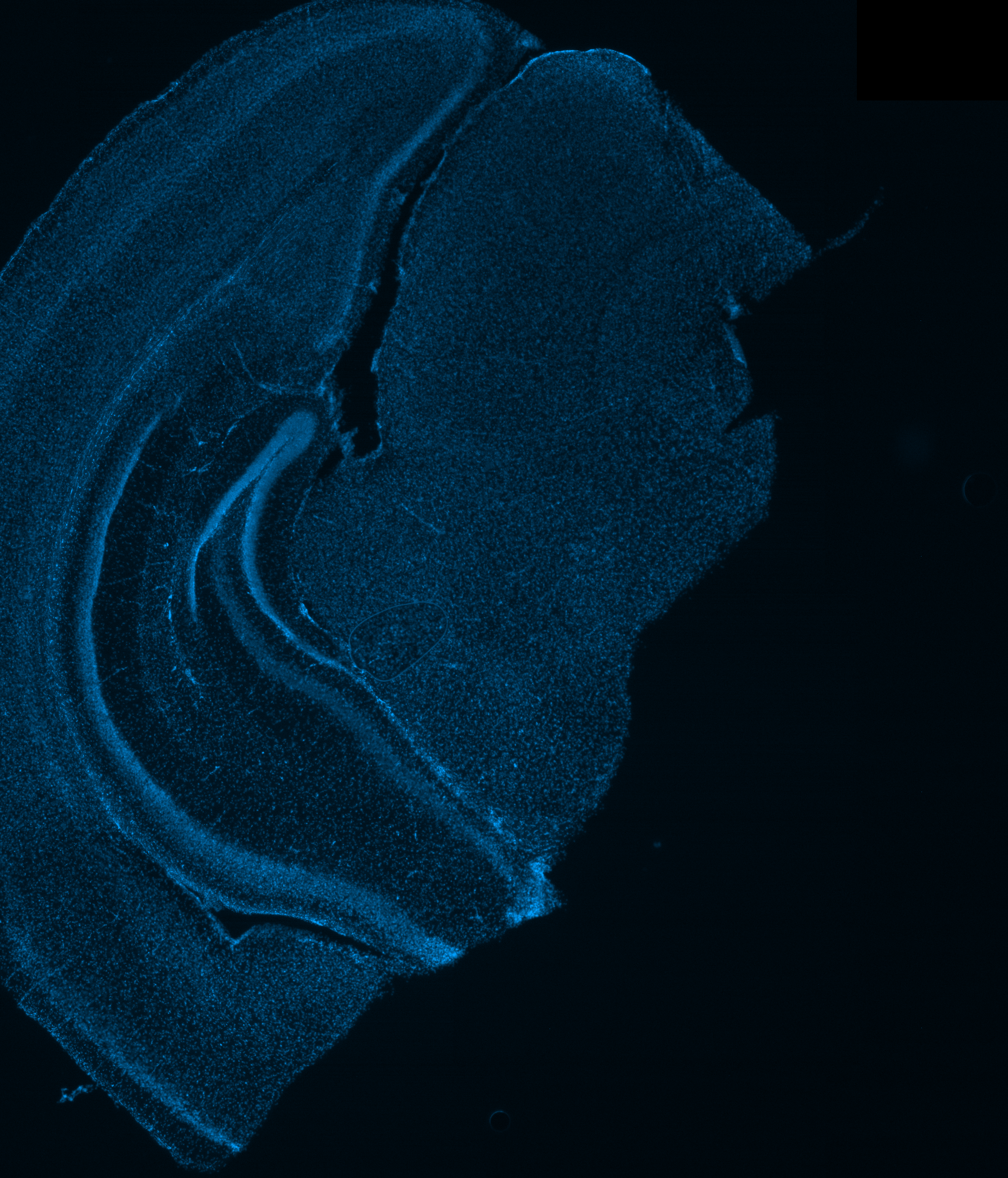

Supplement: Supplementary file 5 — Source data Fig. 3 [file 44319_2025_410_MOESM5_ESM.zip › 3B/Ventral - Hoechst.tif]

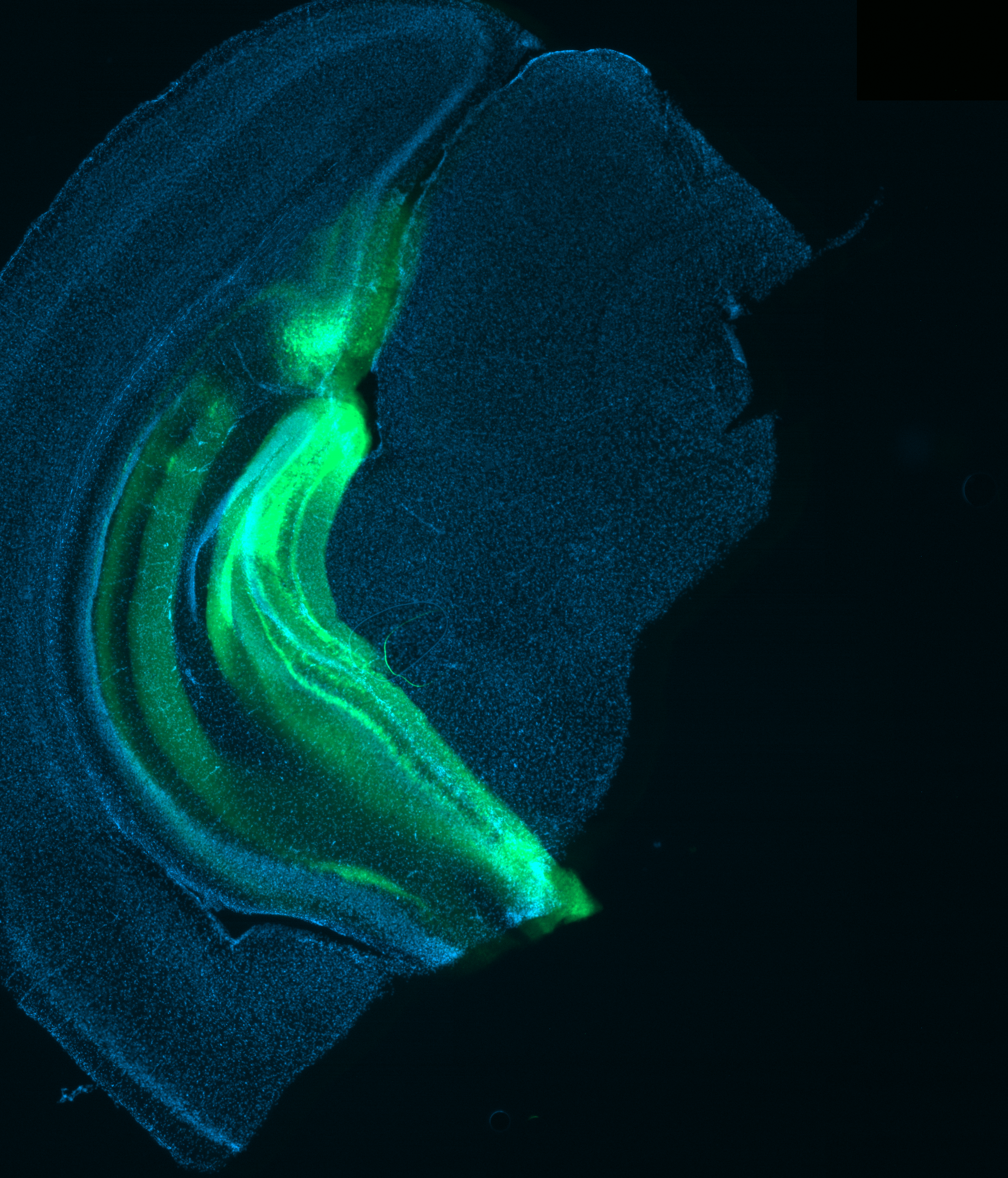

Supplement: Supplementary file 5 — Source data Fig. 3 [file 44319_2025_410_MOESM5_ESM.zip › 3B/Ventral - Merge.tif]

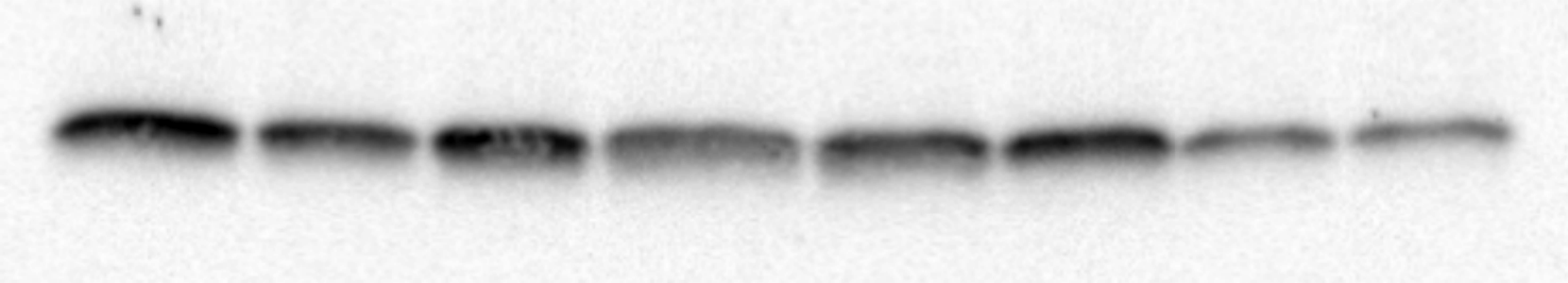

Supplement: Supplementary file 6 — Source data Fig. 4 [file 44319_2025_410_MOESM6_ESM.zip › 4E/Neuronatin cropped full.tif]

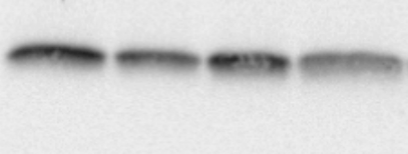

Supplement: Supplementary file 6 — Source data Fig. 4 [file 44319_2025_410_MOESM6_ESM.zip › 4E/Neuronatin cropped.tif]

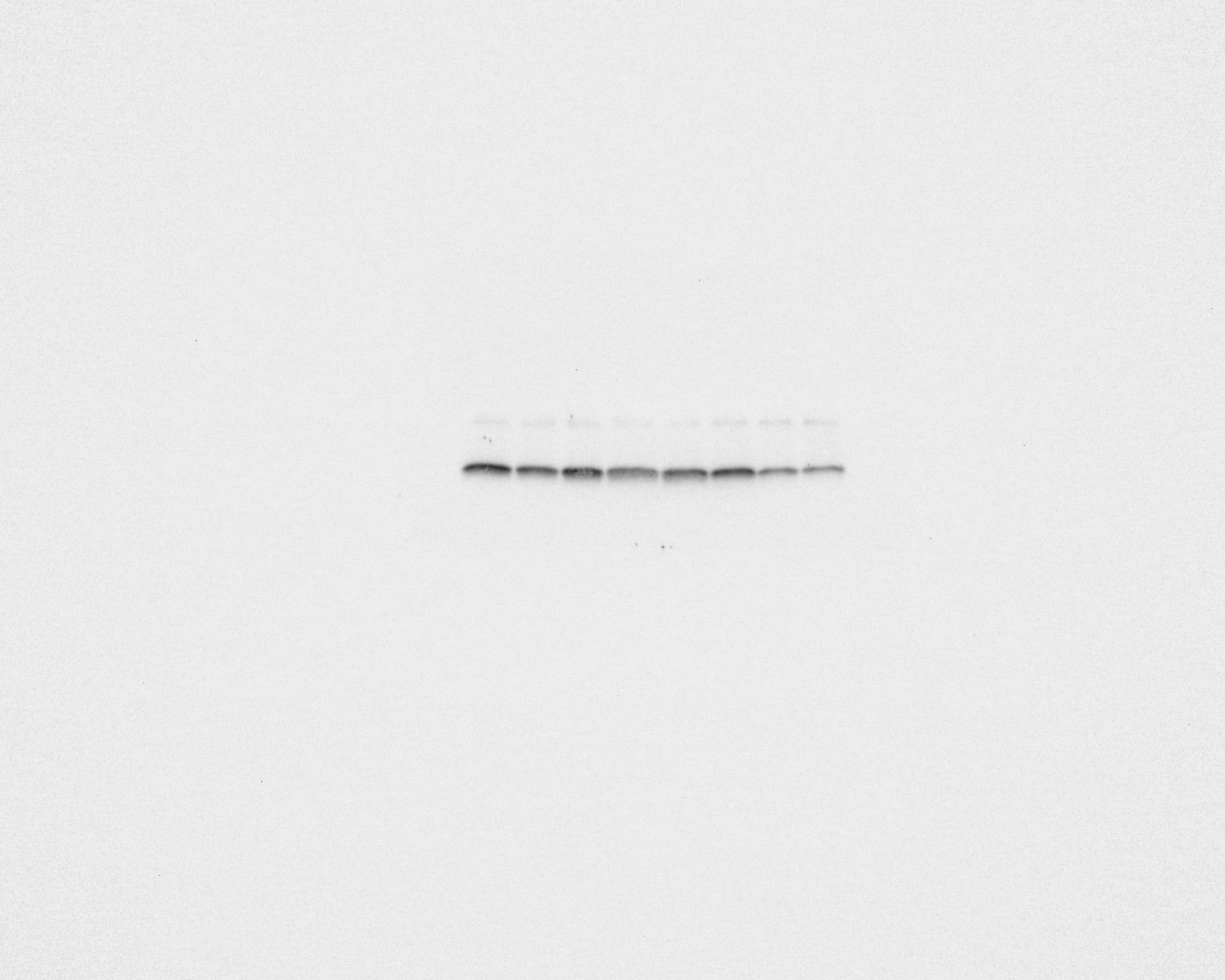

Supplement: Supplementary file 6 — Source data Fig. 4 [file 44319_2025_410_MOESM6_ESM.zip › 4E/Neuronatin.tif]

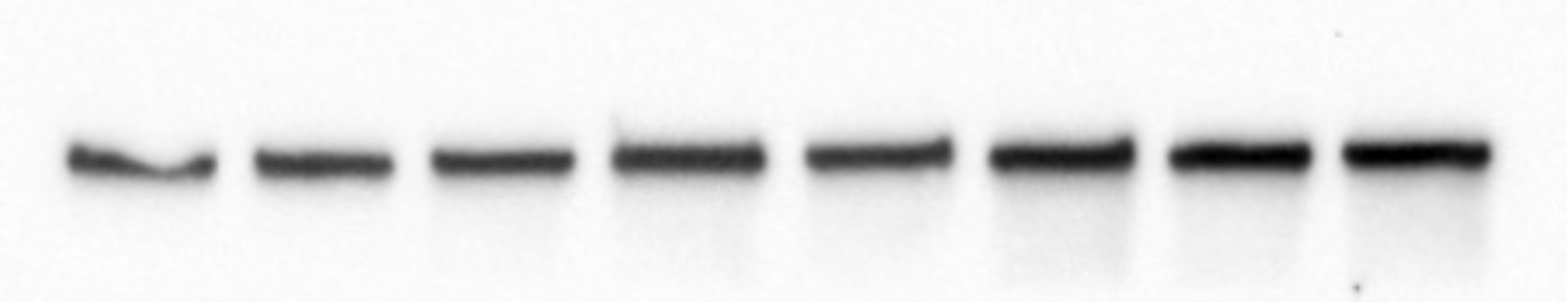

Supplement: Supplementary file 6 — Source data Fig. 4 [file 44319_2025_410_MOESM6_ESM.zip › 4E/Tubulin cropped full.tif]

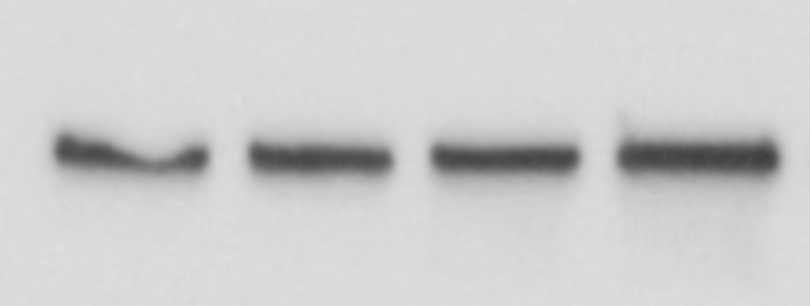

Supplement: Supplementary file 6 — Source data Fig. 4 [file 44319_2025_410_MOESM6_ESM.zip › 4E/Tubulin cropped.tif]

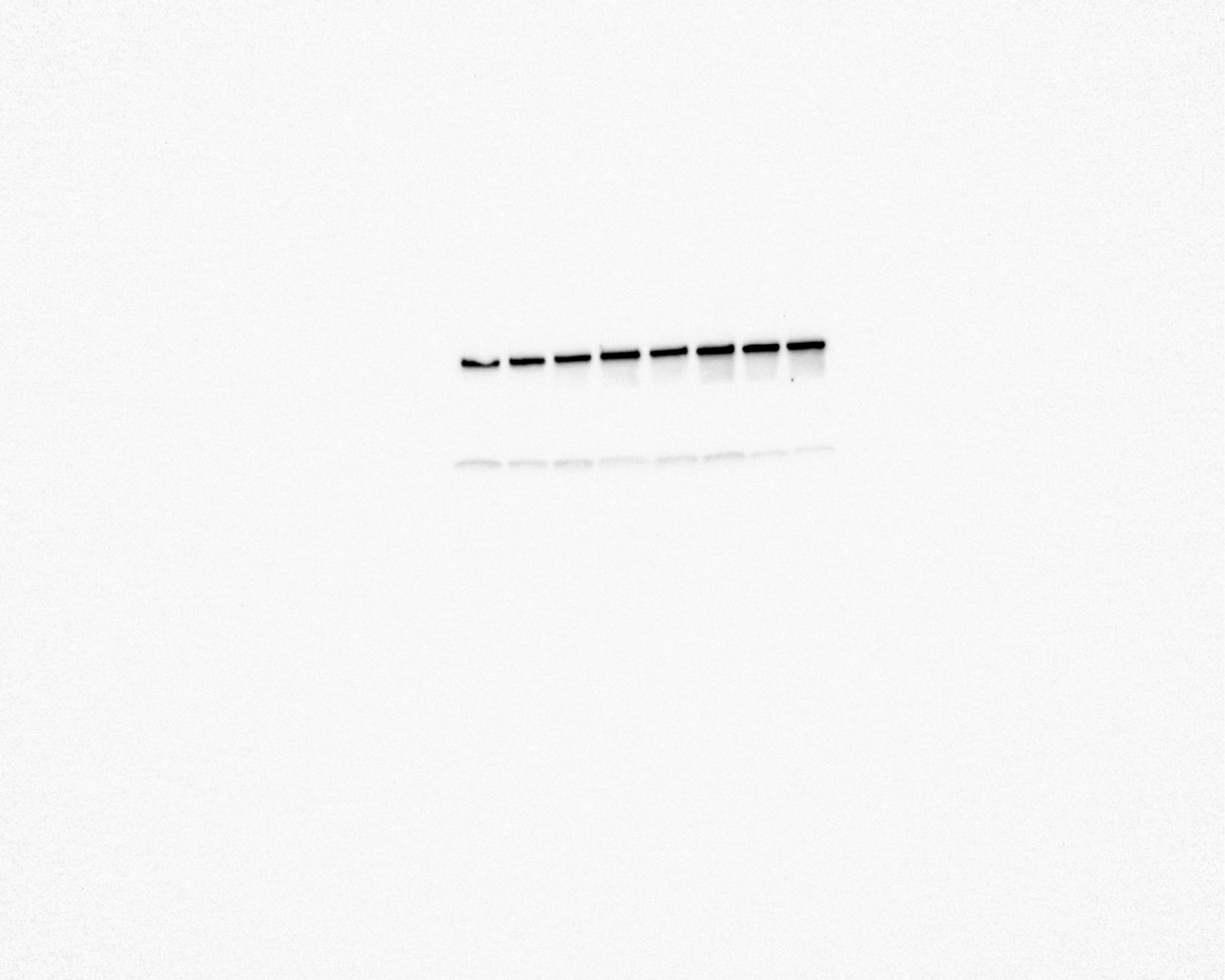

Supplement: Supplementary file 6 — Source data Fig. 4 [file 44319_2025_410_MOESM6_ESM.zip › 4E/Tubulin.tif]

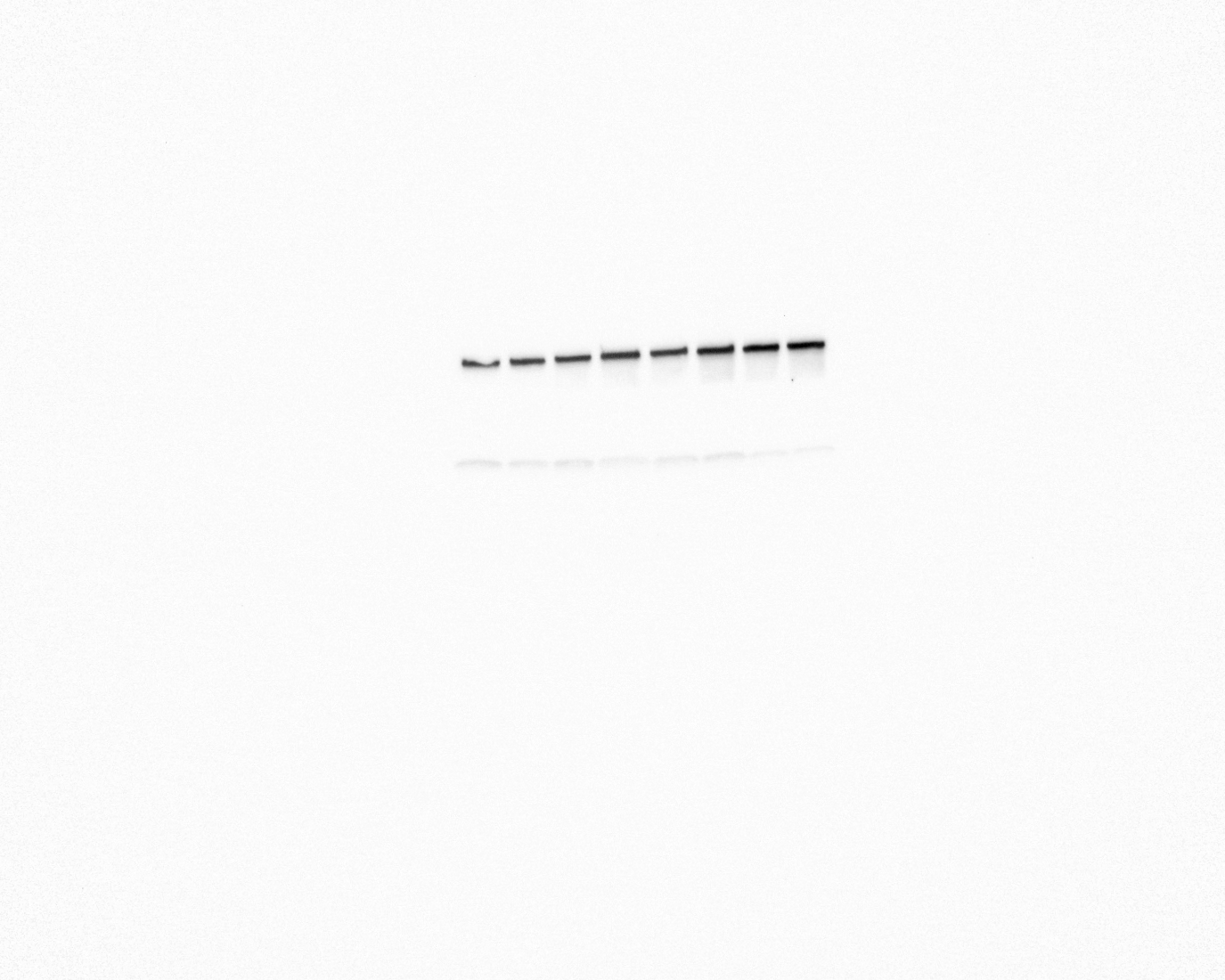

Supplement: Supplementary file 9 — Source Data for Expanded View and Appendix Figures [file 44319_2025_410_MOESM9_ESM.zip › Source Data for Expanded View and Appendix/Expanded View/Figure EV3/EV3C/Tubulin.tif]
