# Supplementary material for: miR-708-5p is elevated in bipolar patients and can induce mood disorder-associated behavior in mice
Source: EMBO Rep. 2025 Mar 10;26(8):2121–45. doi: 10.1038/s44319-025-00410-y (PMC12019553; doi:10.1038/s44319-025-00410-y)
Supplement: Supplementary file 6 — Source data Fig. 4 [file 44319_2025_410_MOESM6_ESM.zip › 4E/README 4E.docx]

**Neuronatin – full membrane**





hp:

708

708

708

CTL

CTL

CTL

708

CTL

**Neuronatin – cropped (Fig. EV3C)**


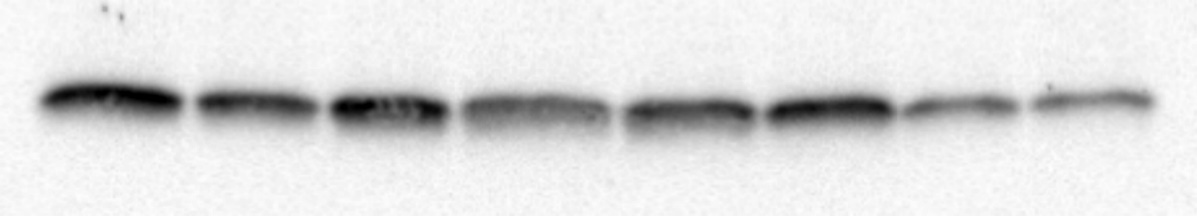


hpCTL hp708 hpCTL hp708 hpCTL hp708 hpCTL hp708

**Neuronatin – cropped (Fig. 4E)**


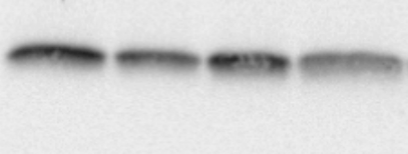


hpCTL hp708 hpCTL hp708

**Tubulin – full membrane**


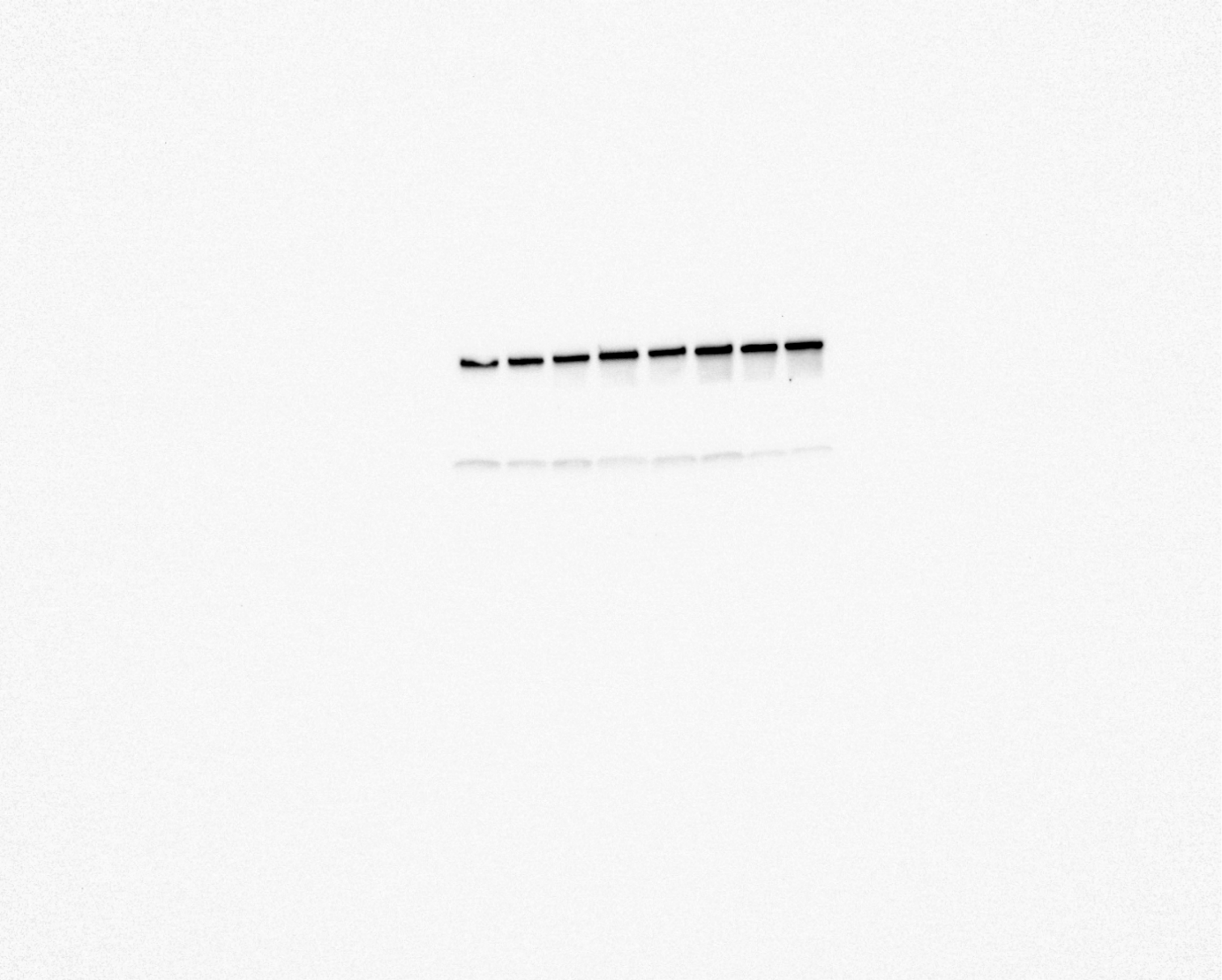


708

708

708

708

CTL

CTL

CTL

CTL

hp:

**Tubulin – cropped (Fig. EV3C)**


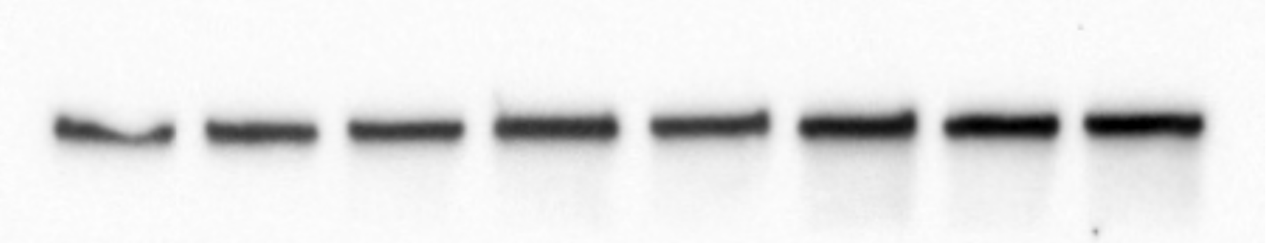


hpCTL hp708 hpCTL hp708 hpCTL hp708 hpCTL hp708

**Tubulin – cropped (Fig. 4E)**


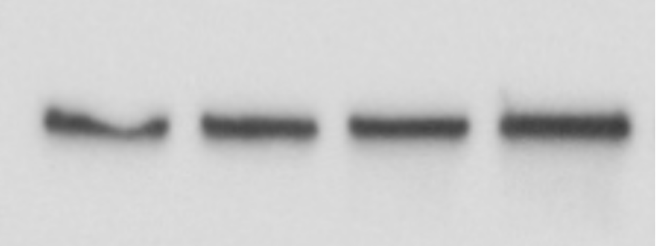


hpCTL hp708 hpCTL hp708
