# Supplementary material for: miR-708-5p is elevated in bipolar patients and can induce mood disorder-associated behavior in mice
Source: EMBO Rep. 2025 Mar 10;26(8):2121–45. doi: 10.1038/s44319-025-00410-y (PMC12019553; doi:10.1038/s44319-025-00410-y)
Supplement: Supplementary file 9 — Source Data for Expanded View and Appendix Figures [file 44319_2025_410_MOESM9_ESM.zip › Source Data for Expanded View and Appendix/Appendix/Appendix Figure S6/S6C-F/S6C-F.html]

Carlotta mir marker


Code 

- Show All Code
- Hide All Code

# Carlotta mir marker

#### Pierre-Luc Germain

#### 2023-05-25

```
knitr::opts_chunk$set(dev = "ragg_png")

suppressPackageStartupMessages({
  library(readxl)
  library(ggplot2)
  library(cowplot)
  library(glmnet)
})
```

```
doEval <- function(ef,val="2^-deltacq"){
  ef <- ef[order(-ef[[val]]),]
  ef$sens <- cumsum(ef$Group=="BD")/sum(ef$Group=="BD")
  ef$spec1 <- cumsum(ef$Group!="BD")/sum(ef$Group!="BD")
  ef$sensVC <- cumsum(ef$Group=="BD")/sum(ef$Group=="BD")
  ef$spec1VC <- cumsum(ef$Group=="Control")/sum(ef$Group=="Control")
  return(ef)
}

rocp <- function(x, facet=~Sex, val="2^-deltacq", vsAll=TRUE, title=NULL){
  x <- as.data.frame(x)[c(1,seq_len(nrow(x))),]
  x[1,c("sens","spec1","spec1VC","sensVC")] <- 0
  if(vsAll){
    if(is.null(title)) title <- "BD vs controls and MDD"
    av <- c("spec1","sens")
  }else{
    if(is.null(title)) title <- "BD vs controls"
    x <- x[x$Group!="MDD",]
    av <- c("spec1VC","sensVC")
  }
  p1 <- ggplot(x, aes_string(av[1],av[2])) +
    geom_abline(slope=1, linetype="dashed", col="grey") + geom_line() + 
    labs(x="1-specificity", y="Sensitivity", title=title) + theme_bw()
  if(!is.null(facet)) p1 <- p1 + facet_wrap(facet)
  if(!is.null(val)){
    if(!is.null(facet)){
      fname <- attr(terms(facet),"term.labels")
      sp <- x[,fname]
      sp <- split(x, sp)
      aucs <- dplyr::bind_rows(lapply(sp, FUN=function(x){
        data.frame(auc=PRROC::roc.curve(x[x$Group=="BD",val],x[x$Group!="BD",val])$auc)
      }), .id=fname)
      th <- dplyr::bind_rows(lapply(sp, FUN=function(x){
        y <- setNames(doThres(x[[av[2]]], x[[av[1]]]),av[2:1])
        y <- as.data.frame(as.list(y))
        y[[fname]] <- x[[fname]][1]
        y
      }))
    }else{
      aucs <- data.frame(auc=PRROC::roc.curve(x[x$Group=="BD",val],x[x$Group!="BD",val])$auc)
      th <- setNames(doThres(x[[av[2]]], x[[av[1]]]),av[2:1])
      th <- as.data.frame(as.list(th))
    }
    aucs$auc <- paste("AUC=",round(aucs$auc,3))
    th$label <- paste0("sens=",round(th[,1],2),"\n","spec=",round(1-th[,2],2))
    p1 <- p1 + geom_point(data=th) + 
      ggrepel::geom_text_repel(data=th, aes(label=label), min.segment.length=0,
                               nudge_y=-0.2, nudge_x=0.2) +
      geom_text(data=aucs, aes(label=auc), x=0.85,y=0.15)
    
  }
  p1
}

doThres <- function(sens,spec1){
  w <- which.min(sqrt((1-sens)^2+(spec1)^2))
  c(sens[w],spec1[w])
}
```

# Mir-708

```
e <- read_excel("carlotta_mir_marker.xlsx", sheet=5)
d <- dplyr::bind_rows(lapply(split(e, e$Sex), FUN=doEval))
plot_grid(rocp(d, ~Sex, val="2^-deltacq"),
          rocp(d, ~Sex, val="2^-deltacq", vsAll=FALSE), nrow=2)
```

```
## Warning: `aes_string()` was deprecated in ggplot2 3.0.0.
## ℹ Please use tidy evaluation idioms with `aes()`.
## ℹ See also `vignette("ggplot2-in-packages")` for more information.
## This warning is displayed once every 8 hours.
## Call `lifecycle::last_lifecycle_warnings()` to see where this warning was
## generated.
```

# Mir-499

```
e2 <- read_excel("carlotta_mir_marker.xlsx", sheet=1)
e3 <- read_excel("carlotta_mir_marker.xlsx", sheet=3)
e2 <- rbind(e2,e3)
d2 <- dplyr::bind_rows(lapply(split(e2, e2$sex), val="2^-deltaCq", FUN=doEval))
plot_grid(rocp(d2, ~sex, val="2^-deltaCq"),
          rocp(d2, ~sex, val="2^-deltaCq", vsAll=FALSE), nrow=2)
```

# Combination

```
e$mir708 <- scale(log2(e$`2^-deltacq`))
e2$mir499 <- scale(log2(e2$`2^-deltaCq`))
m <- merge(e, e2, by.x="ID",by.y="Sample")
m$Sex <- as.integer(m$Sex=="female")
preds <- as.matrix(m[,c("Sex","mir708","mir499")])
set.seed(123)
mod <- cv.glmnet(preds, m$Group.x=="BD", family="binomial", nfolds=3, type.measure="auc", lambda=c(0.0001,0))
m$predicted <- predict(mod, newx=preds)
m$Group <- m$Group.x
m$sum <- m$mir499+m$mir708
```

## Simple sum of scaled values

```
d3 <- dplyr::bind_rows(lapply(split(m, m$sex), val="sum", FUN=doEval))
plot_grid(rocp(d3, ~sex, val="sum"),
          rocp(d3, ~sex, val="sum", vsAll=FALSE), nrow=2)
```

Without splitting sex:

```
d3m <- d3[d3$sex=="Male",]
pdf("malesOnly.sum.ROC.pdf", width=3,height=3)
rocp(d3m, NULL, val="sum") + labs(subtitle="Males only")
dev.off()
```

```
## pdf 
##   2
```

```
d3 <- doEval(m, val="sum")
pdf("sexesTogether.sum.ROC.pdf", width=3,height=3)
rocp(d3, NULL, val="sum") + labs(subtitle="Both sexes together")
dev.off()
```

```
## pdf 
##   2
```

```
plot_grid(rocp(d3, NULL, val="sum"),
          rocp(d3, NULL, val="sum", vsAll=FALSE), nrow=1)
```

## Logistic regression

```
d3 <- dplyr::bind_rows(lapply(split(m, m$sex), val="predicted", FUN=doEval))
plot_grid(rocp(d3, ~sex, val="predicted"),
          rocp(d3, ~sex, val="predicted", vsAll=FALSE), nrow=2)
```

Without splitting sex:

```
d3 <- doEval(m, val="predicted")
plot_grid(rocp(d3, NULL, val="predicted"),
          rocp(d3, NULL, val="predicted", vsAll=FALSE), nrow=1)
```

### Training only on males

```
mm <- m[m$sex=="Male",]
set.seed(123)
preds <- as.matrix(mm[,c("mir708","mir499")])
mod <- cv.glmnet(preds, mm$Group.x=="BD", nfolds=3, family="binomial", type.measure="auc", lambda=c(0.0001,0))
mm$predicted <- predict(mod, newx=preds)
mm$Group <- mm$Group.x
mm <- doEval(mm, val="predicted")
rocp(mm, NULL, val="predicted") + theme(aspect.ratio = 1)
```
