# Supplementary material for: miR-708-5p is elevated in bipolar patients and can induce mood disorder-associated behavior in mice
Source: EMBO Rep. 2025 Mar 10;26(8):2121–45. doi: 10.1038/s44319-025-00410-y (PMC12019553; doi:10.1038/s44319-025-00410-y)
Supplement: Supplementary file 10 — Expanded View Figures [file 44319_2025_410_MOESM10_ESM.pdf]

Expanded View Figures

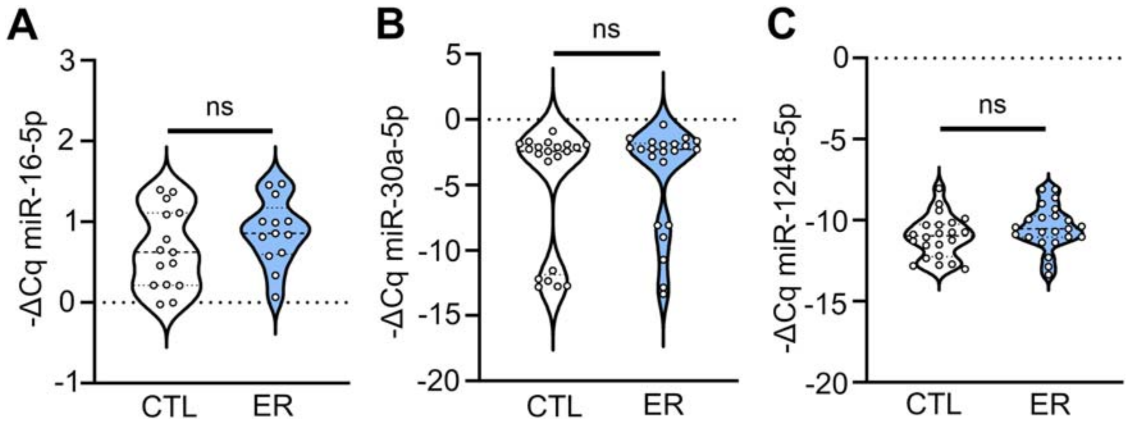

Figure EV1. Unrelated miRNAs tested by qPCR as negative controls for the CTL *vs* ER small-RNA sequencing.

(**A**) miR-16-5p qPCR analysis of total RNA isolated from PBMCs of CTL ($n = 15$) and ER ($n = 13$) subjects. Unpaired t-test, ns. Data are presented as violin plots with median, quartiles and data points. (**B**) miR-30a-5p qPCR analysis of total RNA isolated from PBMCs of CTL ($n = 15$) and ER ($n = 16$) subjects. Unpaired t-test, ns. Data are presented as violin plots with median, quartiles and data points. (**C**) miR-1248-5p qPCR analysis of total RNA isolated from PBMCs of CTL ($n = 16$) and ER ($n = 17$) subjects. Unpaired t-test, ns. Data are presented as violin plots with median, quartiles and data points. Source data are available online for this figure.

© The Author(s)

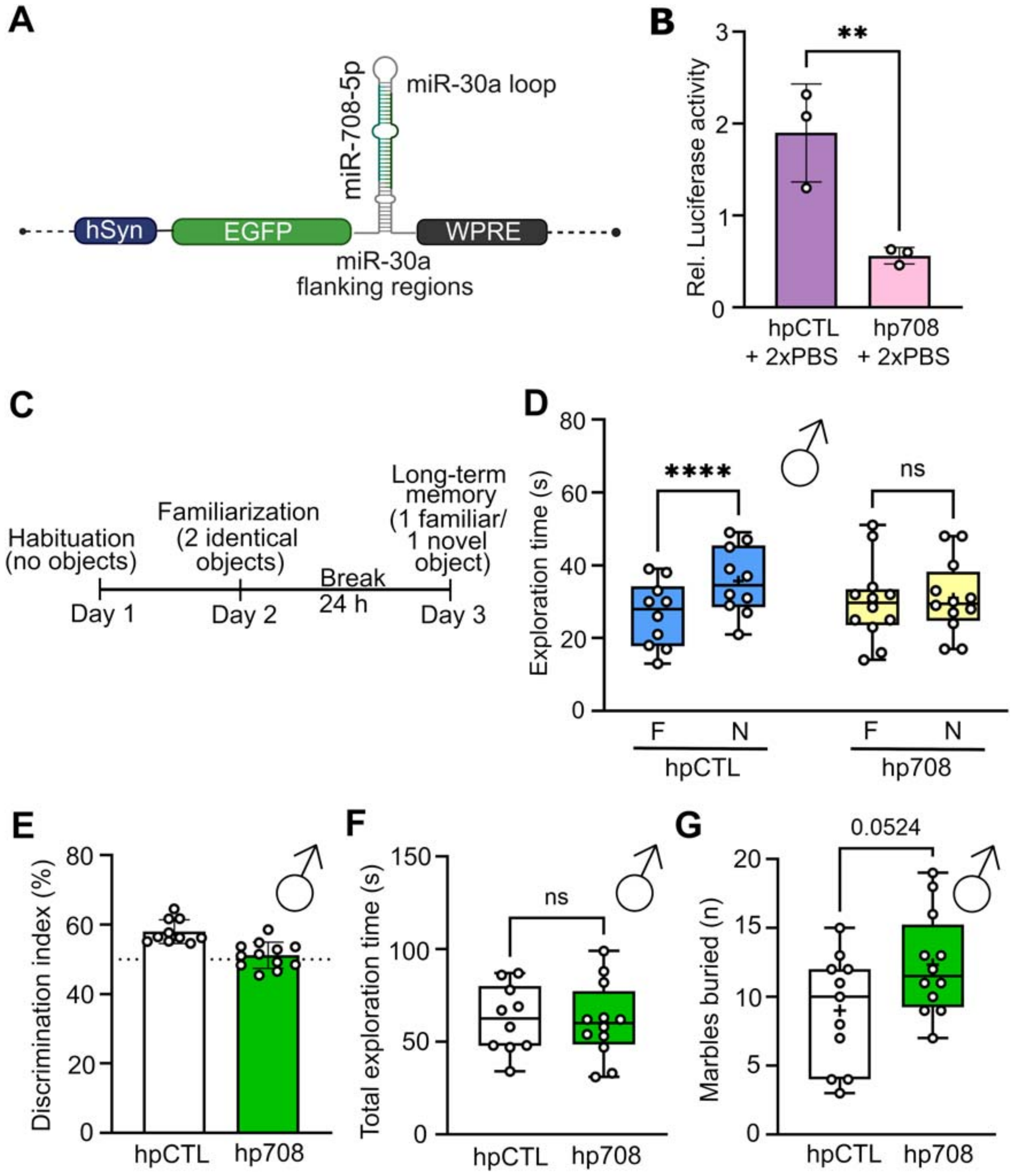

Figure EV2. Total time exploring objects (novel plus familiar) and discrimination index for the novel object test.

Related to Fig. 3. (**A**) Schematic representation of the miR-708-5p overexpressing hairpin (hp708), placed in the 3′-UTR of EGFP. (**B**) Relative luciferase activity of rat hippocampal neurons transfected with the indicated plasmid (hpCTL or hp708) and a luciferase reporter expressing 2x Perfect Binding sites (PBS) for miR-708-5p. Data are represented as scattered dot plots with bar, mean ± SD ($n = 3$ biological replicates). Ratio paired t test, two-tailed, $^{**}p = 0.0057$. (**C**) Schematic representation of the Novel Object Recognition (NOR) test for long-term memory. The break between the familiarization and the long-term memory sessions is of 24 h. (**D**) Time (s) male mice injected with the indicated rAAV (hpCTL $n = 10$, hp708, $n = 12$) explored either the familiar (F) or novel (N) object. Data are represented as box plot with whiskers and data points (+: mean, line: median; whiskers: minimum and maximum values). Two-way RM ANOVA: Novelty × Group, $^{***}p = 0.0004$; Novelty, $^{****}p < 0.0001$; Group, ns, $p = 0.8830$. Šídák's post hoc test, F *vs* N: hpCTL, $^{****}p < 0.0001$; hp708, ns, $p = 0.6735$. (**E**) Discrimination index calculated as time spent exploring novel object/time spent exploring novel and familiar objects for male mice injected with the indicated rAAV (hpCTL $n = 12$, hp708, $n = 11$) in the Novel Object Recognition long-term session. Data are represented as scattered dot plots with bar, mean ± SD. (**F**) Time (s) male mice injected with the indicated rAAV (hpCTL $n = 12$, hp708, $n = 11$) explored the familiar (F) and novel (N) object in the Novel Object Recognition long-term session. Data are represented as box plot with whiskers and data points (+: mean, line: median; whiskers: minimum and maximum values). Unpaired t-test, ns, $p = 0.8869$. (**G**) Number of marbles male mice injected with the indicated rAAV (hpCTL $n = 11$, hp708, $n = 12$) buried in 30 min. Data are represented as box plot with whiskers and data points (+: mean, line: median; whiskers: minimum and maximum values). Unpaired t-test, $p = 0.0524$. Source data are available online for this figure.

 © The Author(s)

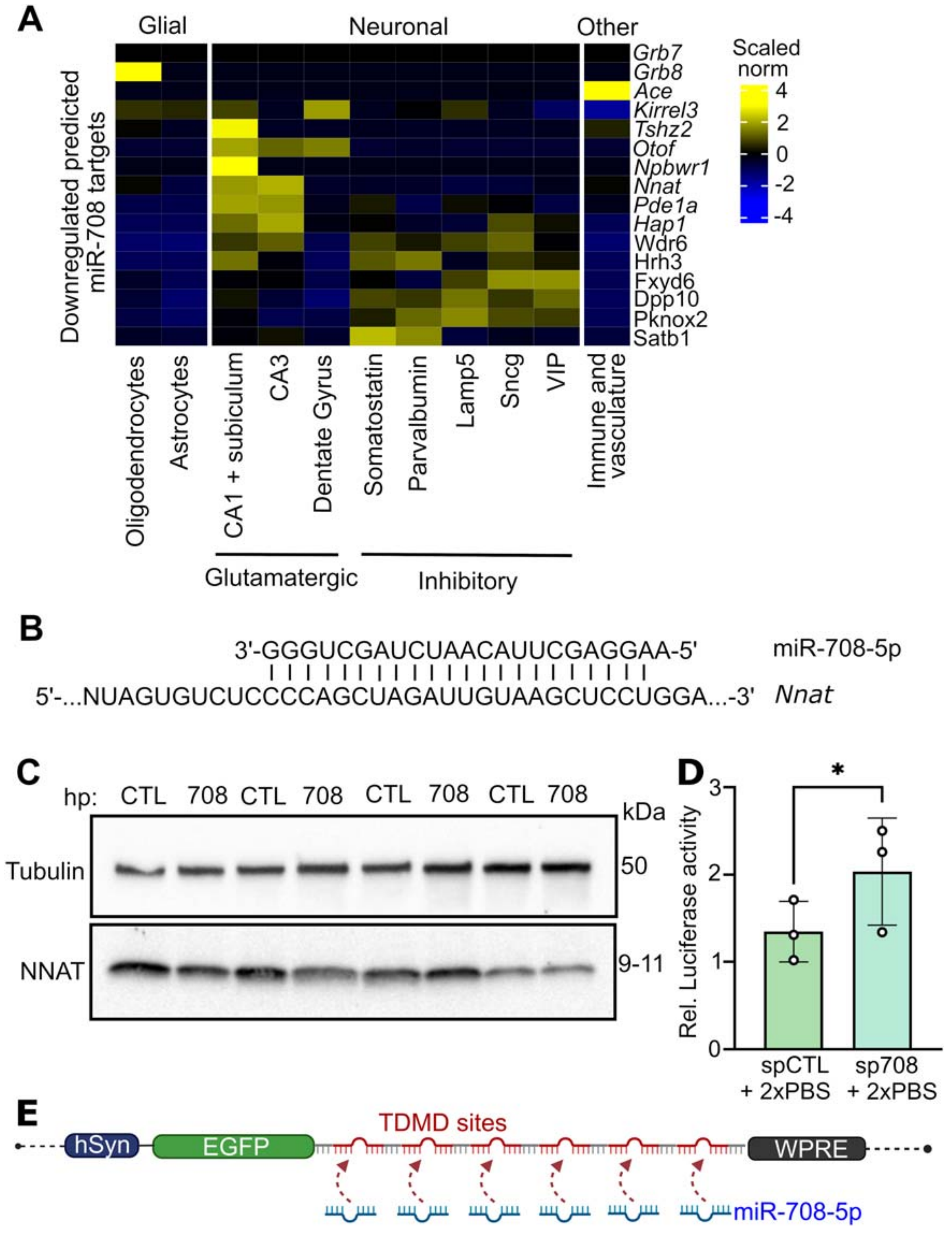

Figure EV3. Validation of *Nnat* as a miR-708-5p target.

Related to Fig. 4. (**A**) Heatmap displaying miR-708-5p predicted targets which are significantly downregulated (from Fig. 4A), as well as their expression in different cell types based on single-cell RNA-seq data (Allen 10X+smartSeq taxonomy). (**B**) Nucleotide base pairing depicting the perfect binding of miR-708-5p with the mouse *Nnat* 3′-UTR. (**C**) Full Western blot image (Fig. 4E) of NNAT (lower panel) and Tubulin (upper panel) protein expression levels in hippocampal neurons (20 days in vitro) that were infected with hpCTL or hp708 at 2 days in vitro. (**D**) Relative luciferase activity of rat hippocampal neurons transfected with the indicated plasmid (sponge control: spCTL, miR-708-5p sponge: sp708) and a luciferase reporter expressing 2x Perfect Binding sites (PBS) for miR-708-5p. Data are represented as scattered dot plots with bar, mean ± SD ($n = 3$ biological replicates). Ratio paired t-test, two-tailed, *$p = 0.0372$. (**E**) Schematic representation of the construct to knock-down miR-708-5p via six TDMD sites, used in Fig. 4G (right) and Fig. EV3D. Source data are available online for this figure.

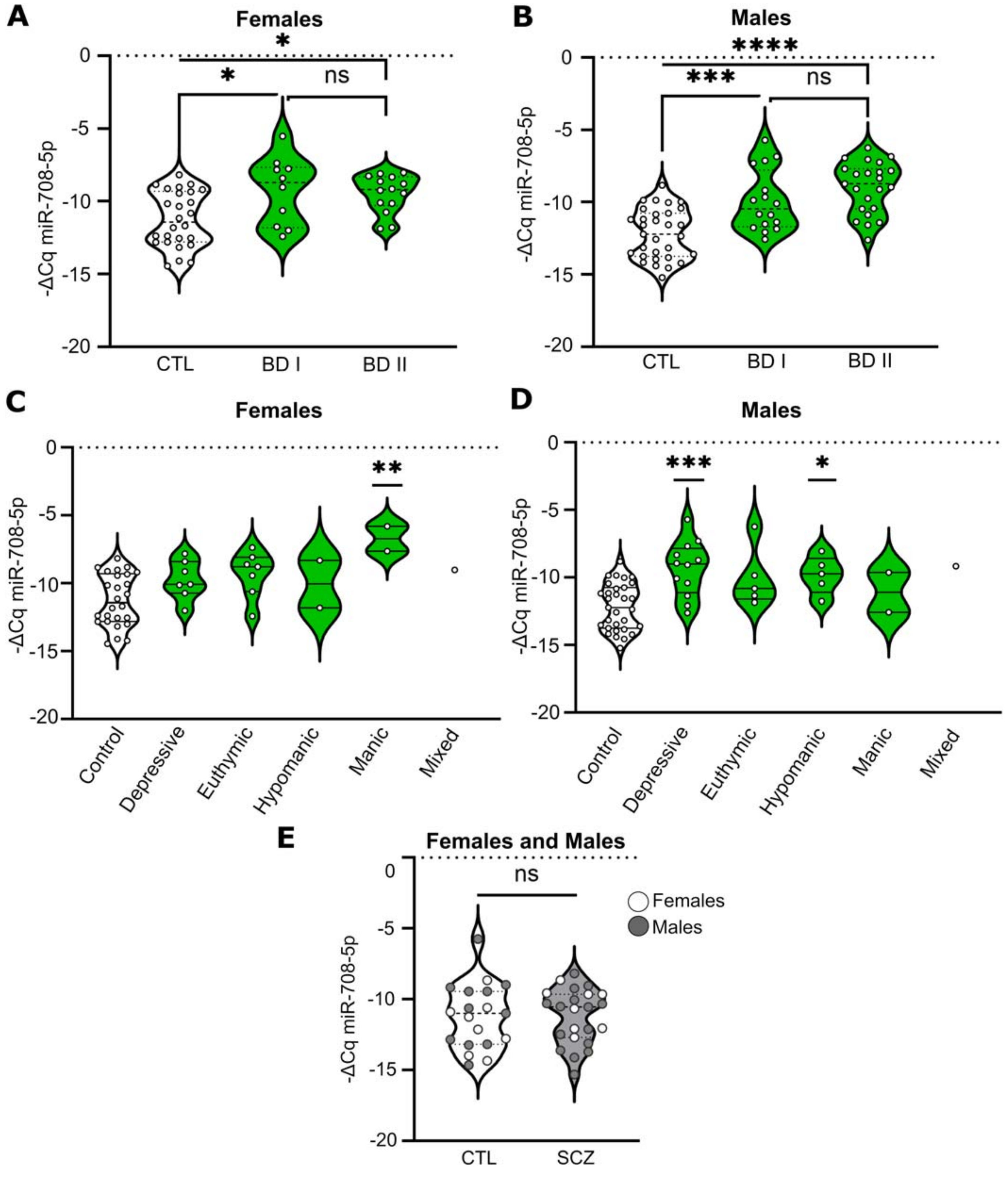
A
Females
*
*
ns
-ΔCq miR-708-5p
0
-5
-10
-15
-20
CTL
BD I
BD II
B
Males
****
***
ns
-ΔCq miR-708-5p
CTL
BD I
BD II
C
Females
**
Control
Depressive
Euthymic
Hypomanic
Manic
Mixed
D
Males
***
*
Control
Depressive
Euthymic
Hypomanic
Manic
Mixed
E
Females and Males
ns
Females
Males
CTL
SCZ

 © The Author(s)

Figure EV4. miR-708-5p expression levels in the PBMCs of subjects with different bipolar types and mood states.

Related to Fig. 6. (**A**) miR-708-5p expression levels in PBMCs of female subjects with Bipolar disorder type I (BD I) or type II (BD II) (CTL, $n = 26$; BD I, $n = 10$; BD II, $n = 14$). Kruskal Wallis Test, Post hoc Dunn's: Control *vs* BD I: *$p = 0.0253$, Control *vs* BD II: *$p = 0.0196$, BD I *vs* BD II: ns, $p > 0.9999$. Data are presented as violin plots with median, quartiles, and data points. (**B**) miR-708-5p expression levels in PBMCs of male subjects with BD I or BD II (CTL $n = 31$; BD I, $n = 16$; BD II, $n = 23$). One-way ANOVA, Post hoc Tukey's test: Control *vs* BD I: ***$p = 0.0005$, Control *vs* BD II: ****$p = 0.0001$, BD I *vs* BD II: ns, $p = 0.2690$. Data are presented as violin plots with median, quartiles, and data points. (**C**) miR-708-5p qPCR analysis of total RNA isolated from PBMCs of healthy control female subjects (control, $n = 26$) and BD subjects in different mood states (depressive, $n = 7$; euthymic, $n = 7$; hypomanic, $n = 25$; manic, $n = 2$; mixed, $n = 1$). Kruskal Wallis Test, Post hoc Dunn's: Control *vs* Manic, *$p = 0.0300$. Data are presented as violin plots with median, quartiles, and data points. (**D**) miR-708-5p qPCR analysis of total RNA isolated from PBMCs of healthy control male subjects (control, $n = 31$) and BD subjects in different mood states (depressive, $n = 12$; euthymic, $n = 5$; hypomanic, $n = 5$; manic, $n = 2$; mixed, $n = 1$). One-way ANOVA, Post hoc Dunnett's test: Control *vs* Depressive, ***$p = 0.0002$; Control *vs* Hypomanic, *$p = 0.0490$. Data are presented as violin plots with median, quartiles, and data points. (**E**) miR-708-5p qPCR analysis of total RNA isolated from PBMCs of male and female patients diagnosed with Schizophrenia (male: CTL $n = 11$, SCZ $n = 15$; female: CTL $n = 8$, SCZ $n = 8$). Two-way ANOVA: Group × Sex, ns, $p = 0.4904$; Group, ns, $p = 0.2383$, Sex, ns, $p = 0.4904$. Tukey's post hoc test, ns. Data are presented as violin plots with median, quartiles, and data points. Source data are available online for this figure.

© The Author(s)
